# The Tau Tubulin Kinases TTBK1/2 Promote Accumulation of Pathological TDP-43

**DOI:** 10.1371/journal.pgen.1004803

**Published:** 2014-12-04

**Authors:** Nicole F. Liachko, Pamela J. McMillan, Timothy J. Strovas, Elaine Loomis, Lynne Greenup, Jill R. Murrell, Bernardino Ghetti, Murray A. Raskind, Thomas J. Montine, Thomas D. Bird, James B. Leverenz, Brian C. Kraemer

**Affiliations:** 1 Geriatric Research Education and Clinical Center, Veterans Affairs Puget Sound Health Care System, Seattle, Washington, United States of America; 2 Department of Medicine, University of Washington, Seattle, Washington, United States of America; 3 Mental Illness Research Education and Clinical Center, Veterans Affairs Puget Sound Health Care System, Seattle, Washington, United States of America; 4 Department of Psychiatry and Behavioral Sciences, University of Washington, Seattle, Washington, United States of America; 5 Department of Pathology & Laboratory Medicine, Indiana University School of Medicine, Indianapolis, Indiana, United States of America; 6 Department of Neurology, University of Washington, Seattle, Washington, United States of America; 7 Parkinson's Disease Research Education and Clinical Center, Veterans Affairs Puget Sound Health Care System, Seattle, Washington, United States of America; 8 Department of Pathology, University of Washington, Seattle, Washington, United States of America; Baylor College of Medicine, United States of America

## Abstract

Pathological aggregates of phosphorylated TDP-43 characterize amyotrophic lateral sclerosis (ALS) and frontotemporal lobar degeneration (FTLD-TDP), two devastating groups of neurodegenerative disease. Kinase hyperactivity may be a consistent feature of ALS and FTLD-TDP, as phosphorylated TDP-43 is not observed in the absence of neurodegeneration. By examining changes in TDP-43 phosphorylation state, we have identified kinases controlling TDP-43 phosphorylation in a *C. elegans* model of ALS. In this kinome-wide survey, we identified homologs of the tau tubulin kinases 1 and 2 (TTBK1 and TTBK2), which were also identified in a prior screen for kinase modifiers of TDP-43 behavioral phenotypes. Using refined methodology, we demonstrate TTBK1 and TTBK2 directly phosphorylate TDP-43 *in vitro* and promote TDP-43 phosphorylation in mammalian cultured cells. TTBK1/2 overexpression drives phosphorylation and relocalization of TDP-43 from the nucleus to cytoplasmic inclusions reminiscent of neuropathologic changes in disease states. Furthermore, protein levels of TTBK1 and TTBK2 are increased in frontal cortex of FTLD-TDP patients, and TTBK1 and TTBK2 co-localize with TDP-43 inclusions in ALS spinal cord. These kinases may represent attractive targets for therapeutic intervention for TDP-43 proteinopathies such as ALS and FTLD-TDP.

## Author Summary

Aggregated proteins are a hallmark of many neurodegenerative diseases. In ALS and FTLD-TDP, these aggregates contain abnormal TDP-43 modified by phosphorylation. Protein phosphorylation normally controls protein activity, stability, or location, but in some neurodegenerative diseases the phosphorylated proteins accumulate in excess. Kinases are the enzymes responsible for protein phosphorylation. We have identified two TDP-43 kinases, TTBK1 and TTBK2, using a novel approach combining reverse genetics and biochemical screening to identify the kinases responsible for changes in TDP-43 phosphorylation. We show TTBK1 and TTBK2 directly phosphorylate TDP-43 *in vitro*, and control TDP-43 phosphorylation in cellular and simple animal models of ALS. This has uncovered a molecular mechanism by which pathological phosphorylated TDP-43 can occur in disease. To determine whether changes in TTBK1/2 protein are contributing to TDP-43 pathology, we examined diseased brain and spinal cord tissue from patients with ALS or FTLD-TDP. We observed changes in the abundance of TTBK1 and TTBK2 in disease-affected neurons, and the coexistence of TTBK1/2 with phosphorylated TDP-43 aggregates in both FTLD-TDP and ALS. Therefore, increased abundance or activity of TTBK1 or TTBK2 may contribute to the neurodegeneration observed in ALS and FTLD-TDP.

## Introduction

Ubiquitinated, hyperphosphorylated inclusions of the protein TDP-43 characterize disease-affected neurons in patients with amyotrophic lateral sclerosis (ALS) and frontotemporal lobar dementia (FTLD-TDP) [1],[2]. Mutations in the human gene coding for TDP-43, *TARDBP*, were found to cause ALS in a subset of affected families, supporting a causal role for TDP-43 in disease initiation [3]–[7]. In addition to being the hallmark lesions in ALS and FTLD-TDP, inclusions containing TDP-43 are varyingly present in some other neurodegenerative diseases, including Alzheimer's disease (AD), Parkinson's disease, dementia with Lewy bodies, Huntington's disease, and chronic traumatic encephalopathy (CTE) [8]–[12], where the severity of TDP-43 pathologic change is associated with the rate of cognitive decline in affected patients [13]. Many model systems including *C. elegans*, *Drosophila*, zebrafish, mice, and rats have demonstrated neurotoxicity resulting from mutant TDP-43 [14]–[19]. Therefore, TDP-43 pathologic change is not merely a hallmark of disease, but TDP-43 dysfunction can cause neurodegeneration.

TDP-43 undergoes a number of pathological modifications in disease-affected neurons including ubiquitination, phosphorylation, and proteolytic processing. These modifications may promote aggregation and the formation of detergent-insoluble inclusions. The precise molecular cause underlying neurotoxicity in most TDP-43 proteinopathies remains unclear, although the toxicity of mutant TDP-43 expressed in multiple model systems indicates it may be acting through a gain-of-function mechanism via aberrant interactions with proteins and/or nucleic acids [20]. Phosphorylation is a robust and consistent hallmark of pathological TDP-43, and detection of phosphorylation at tandem serines 409 and 410 characterizes virtually all TDP-43 proteinopathy cases [21], [22].

In order to investigate the causes driving pathological TDP-43 phosphorylation, we have developed a *C. elegans* model of TDP-43 proteinopathy exhibiting TDP-43 phosphorylation dependent neurodegeneration and neurotoxicity; in *C. elegans*, phosphorylation of TDP-43 at serines 409 and 410 suffices to promote TDP-43 mediated neurotoxicity [14]. Further, we have used the model to previously identify the kinase CDC7 as a direct modulator of TDP-43 motor phenotypes [23]. This work also showed multiple kinases regulate TDP-43 phosphorylation in *C. elegans*, because detectable phosphorylated TDP-43 remains in the absence of CDC7. Inhibition of the kinases CDC7 or CK1 has also been shown to reduce but not eliminate TDP-43 phosphorylation in cultured cells [23], [24]. Here we utilize the direct detection of changes in TDP-43 phosphorylation by immunoblot analysis of TDP-43 phosphorylation state to discover additional TDP-43 kinases in *C. elegans*. We have identified homologs of the tau tubulin kinases TTBK1 and TTBK2 and characterized their function as regulators of TDP-43 phosphorylation. TTBK1/2 may be attractive drug targets for therapeutic interventions in TDP-43 proteinopathies such as FTLD-TDP and ALS.

## Results

### RNAi screen for TDP-43 kinases controlling pS409/410 TDP-43 levels

To identify TDP-43 kinases, we undertook a comprehensive survey utilizing kinase-targeting RNAi coupled with direct immunoblot detection of changes in TDP-43 phosphorylation in *C. elegans*. We have assembled an RNAi library targeting 451 predicted kinase genes in *C. elegans* (95% coverage of the predicted kinases found in the *C. elegans* genome, Table S1). This library has been previously employed to identify kinase modifiers of TDP-43 dependent behavioral phenotypes, and identified CDC7 as a direct TDP-43 kinase responsible for promoting TDP-43 neurotoxicity [23]. However, CDC7 is not solely responsible for the phosphorylation observed in our *C. elegans* model as detectable phosphorylation at S409/410 is still observed in a *cdc-7(−/−)* null mutant background. Thus other kinases play conserved roles phosphorylating TDP-43, and previous behavior-based screening may have failed to uncover kinases with multiple roles *in vivo*, or kinases whose loss of function could adversely impact motor function or viability independent of TDP-43. To identify additional TDP-43 kinases, a direct biochemical assay of TDP-43 phosphorylation in TDP-43 transgenic *C. elegans* was used to screen for alterations in pS409/410 TDP-43 phosphorylation. Populations of transgenic *C. elegans* expressing ALS-mutant M337V TDP-43 were grown on bacteria producing double stranded RNA targeting each kinase, then harvested and tested by immunoblot for changes in TDP-43 phosphorylation (S1 Figure). Transgenic *C. elegans* expressing ALS mutant TDP-43 exhibit post-translational modification of TDP-43 including prominent phosphorylation [14] in addition to altered proteolytic processing and ubiquitination. Candidate TDP-43 modifying kinases were selected whose knockdown by RNAi robustly reduced the observed TDP-43 phosphorylation relative to control treated animals. Apparent hits were retested by RNAi and immunoblot to confirm decreased TDP-43 phosphorylation, and the identity of positive RNAi clones was confirmed by direct DNA sequencing. Candidate kinases with human homologs acting on serine and/or threonine residues (S/T) were selected for further analysis. A total of 7 candidate S/T kinases were identified that consistently decreased TDP-43 S409/410 phosphorylation following RNAi treatment (Table 1). Interestingly, two of these kinases, *cdc-7* and *mlk-1*, were identified previously in behavior-based screening for TDP-43 kinases [23]. Behavior-based screening also identified three additional homologs of the mammalian tau tubulin kinases TTBK1 and TTBK2, in the CK1 group. The CK1 group of kinases has greatly expanded in *C. elegans*, from 12 members in humans to 86 members in *C. elegans*, including 32 TTBK and TTBKL (TTBK-like) family members [25]. The dramatic expansion of the CK1 family of kinases in *C. elegans* suggests a diversification of functional roles for the TTBK1/2 like kinases in the nematode.

**Table 1:** Candidate TDP-43 kinases identified by RNAi screening.

*C. elegans* Gene (A)	Human Homolog (B)	Kinase Family (C)	Group (C)	#ID'd/# in Kinome (D)	Putative Function (E)	Mutant (F)	Decreased phos (G)
***cdc-7***	CDC7	CDC7	Other	(1/1)	Regulates S-phase and chromatin assembly, acts in DNA replication and damage response	tm4391	**yes**
**H05L14.1**	TTBK1/2	Dual	CK1	(1/3)	Phosphorylates tau; TTBK2 mutation causes SCA11; TTBK1 associated with AD	tm4720	**yes**
***dkf-2***	PRKD2/3	PKD	CAMK	(1/2)	Regulates innate immunity	tm4076	**yes**
***nsy-1***	MAP3K5	STE11	STE	(1/2)	Regulates pathogen response	ok593	no
***kin-20***	CSNK1D	CK1	CK1	(1/1)	Protein complex assembly; mRNA is upregulated in Alzheimer disease	ok505	no
***mlk-1***	MAP3K9	MLK	TKL	(1/2)	Activates JNK signaling cascade; important for axonal regeneration and neuronal apoptosis	ok2471, km19	no
**F39F10.3**	CSNK1A1L	Worm8	CK1	(1/3)	Inositol phosphate-mediated and GPCR signaling, protein binding; associated with 5q syndrome and Alzheimer's disease	tm4396	no

(A) Kinase suppressors of TDP-43 phosphorylation, identified by RNAi.

(B) The human homologs of *C. elegans* genes are the best candidates identified by BLAST protein analysis (HUGO gene nomenclature).

(C) *C. elegans* kinases are assigned to a kinase family and group based on protein sequence analysis [50].

(D) The number of kinase family members identified as TDP-43 suppressors is compared to the total number of kinases within that family.

(E) Some of the known functions of the human kinase genes are highlighted.

(F) Deletion mutant alleles available for *C. elegans* kinases.

(G) Kinase mutants were tested for changes in TDP-43 phosphorylation by immunoblot.

N/A: not tested.

**Figure 1:**
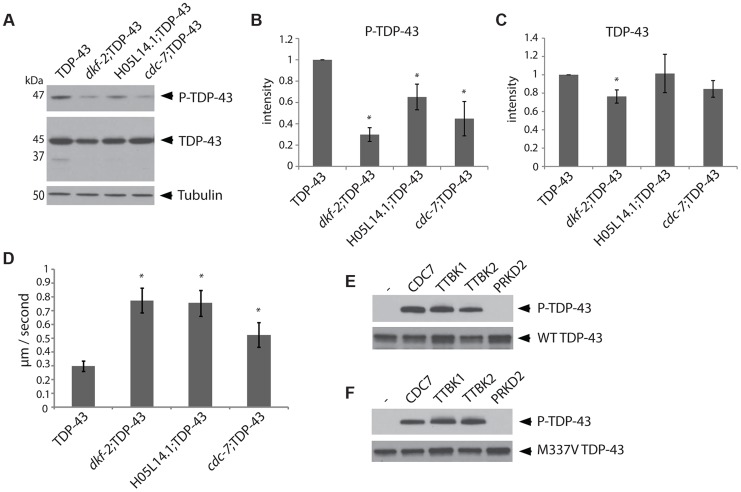
The kinases TTBK1/2 phosphorylate TDP-43 in *C. elegans* and *in vitro*. (A) Developmentally synchronized day 1 adult *dkf-2(−/−)*;TDP-43, *cdc-7(−/−)*;TDP-43, and H05L14.1(−/−);TDP-43 kinase mutants have decreased phosphorylated TDP-43 relative to TDP-43 transgenic animals alone. See S2 Figure for overexposure of immunoblots. Measurement of protein levels of three independent immunoblots is presented for phospho-TDP-43 (B) and total TDP-43 (C). Signal is normalized to the parental TDP-43 transgenic control strain, and graphs are plotted in arbitrary units of intensity. * *P*<0.05, Student's t-test relative to TDP-43 transgenic control. (D) Developmentally staged kinase mutant/TDP-43 transgenic L4 larvae exhibit significantly higher dispersal velocity relative to TDP-43 transgenic animals with intact kinase genes. Animals were measured for the linear distance traveled from a central reference point over time, N>70 for each genotype. *P<0.05 versus TDP-43. Non-transgenic animals disperse at an average velocity of 5.9 µm/second. (E) *In vitro* kinase assays testing the kinase activity of TTBK1, TTBK2, and PRKD2 against wild-type TDP-43 demonstrate purified TTBK1 and TTBK2 phosphorylate wild-type TDP-43, while PRKD2 does not. Immunoblots are probed with antibodies for phosphorylated (P-TDP-43) and total TDP-43. (F) *In vitro* kinase assays demonstrate purified TTBK1 and TTBK2 but not PRKD2 phosphorylate M337V mutant TDP-43. See S4 Figure for controls of kinase activity on known protein substrates.

RNAi can inactivate multiple genes simultaneously depending on their sequence similarity, potentially confounding the identification of any single gene responsible for TDP-43 phosphorylation. To unambiguously determine the effects of single kinase gene loss of function on TDP-43 phosphorylation, we generated TDP-43 transgenic animals with viable deletion mutants eliminating the kinase active domain of each candidate gene of interest (Table 1). Each of these kinase mutants was tested for changes in the amount of phosphorylated TDP-43 by immunoblot. Three of the kinase loss of function mutations tested, *cdc-7(−/−)*, H05L14.1(−/−), and *dkf-2(−/−)*, dramatically reduce TDP-43 phosphorylation with only moderate or no changes in total levels of TDP-43, consistent with the results from the initial RNAi screen (Fig. 1A–C and S2 Figure). We observed a slight decrease in levels of a shorter 37 kDa isoform of TDP-43 (Fig. 1A), but the appearance of higher or lower molecular weight species, including multimers, post-translationally modified protein species, or translational variants, appears relatively unchanged (see S2 Figure for the full α-TDP-43 immunoblot), and after quantitation, only *dkf-2(−/−)* exhibited significant differences in total TDP-43 levels. *cdc-7(−/−)* has been previously characterized as a TDP-43 kinase [23], but we are including analysis of its mutant phenotypes in Fig. 1 for comparison with H05L14.1(−/−) and *dkf-2(−/−)*.

Changes in TDP-43 transgenic animal locomotion can be used as a sensitive measure of TDP-43 toxicity to motor neurons. In fact, we observe that the *cdc-7(−/−)*;TDP-43, H05L14.1(−/−);TDP-43 or *dkf-2(−/−)*;TDP-43 had a more natural and vigorous movement profile relative to the TDP-43 transgene alone. We assessed motor function by measuring the average dispersal velocity of the animals, and found significant improvements compared to TDP-43 (Fig. 1D). These results are consistent with the hypothesis that phosphorylation at S409/410 promotes TDP-43 toxicity, and decreased phosphorylation of TDP-43 will ameliorate the deleterious motor effects resulting from pathological TDP-43.

### TTBK1/2 and PRKD2/3 are human homologs of TDP-43 kinases

To identify human homologs of H05L14.1 and *dkf-2*, we performed an unbiased search for related proteins from eukaryotes within the phylum chordata, including all vertebrate animals. This search employed a basic local alignment search tool (BLAST) [26], followed by automated construction of a phylogenetic tree with the top 50 hits from the search (S3A Figure) [27]. H05L14.1 is related to the human kinase TTBK1, although it is one of many members from an expanded family in *C. elegans* and other ecdysozoa. The H05L14.1 kinase domain has 40% sequence identity to the highly homologous tau tubulin kinases TTBK1 and TTBK2 at the amino acid level (S3C, D Figure) [28]. Variants in the gene coding for TTBK1 are associated with Alzheimer's disease, while mutation in TTBK2 causes spinocerebellar ataxia 11 (SCA11), both of which are characterized by pathologic alterations of tau [26]–[28]. *dkf-2* is related to the conserved protein kinase D family, and is the major representative of the family in *C. elegans* (S3B Figure). The *dkf-2* kinase domain has greater than 70% sequence identity to protein kinase D2 and D3 (PRKD2 and PRKD3) (S3E, F Figure). PRKD2/3 may be involved in cell proliferation, and *dkf-2* has been shown to regulate *C. elegans* innate immunity (Table 1) [29], [30]. Interestingly, our previous search for TDP-43 kinases identified another *C. elegans* homolog of TTBK1/2 [23]. This kinase, C55B7.10 also decreased TDP-43 phosphorylation and improved locomotion in *C. elegans*, although we were unable to determine a direct relationship between human TTBK1/2 and TDP-43 at that time. However, since our last study, we learned TTBK1/2 require millimolar levels of bivalent metal ions Mg^2+^ or Mn^2+^ in the reaction buffer for effective kinase activation [31]. We changed our *in vitro* kinase assay buffer composition, optimizing the reaction conditions for TTBK1/2 kinase assays. The quality of purified TTBK1/2 kinases also affects their activity *in vitro*. We compared purified TTBK1/2 from different commercial sources side by side in an *in vitro* kinase assay against a known target, tau, and found major differences in kinase activity (S4A Figure). Our previous characterization of TTBK1/2 as potential TDP-43 kinases used commercially available purified kinase with low activity against tau. Switching to a more active kinase preparation and modifying the buffer composition in the assay allowed a re-assessment of these potential TDP-43 kinases *in vitro*.

### Human TTBK1/2 directly phosphorylate TDP-43

TDP-43 kinases may act directly by phosphorylating TDP-43 S409/410 or may act indirectly by regulating the activity of other direct TDP-43 kinases. The amino acid sequence in the C-terminus of TDP-43 near S409/410 is consistent with the known CK1 family kinase consensus sequence S/TpXXS/T [32]. The PRKD kinase consensus sequence LXRXMSXXSFX [33], does not conform well with the sequence of human TDP-43. To empirically determine whether human TTBK1/2 or PRKD2/3 are direct TDP-43 kinases, we tested the ability of purified active kinase enzymes to phosphorylate TDP-43 at S409/410 and S403/404 *in vitro* (Fig. 1E, F, S4B Figure). We found that TTBK1 and 2 can directly phosphorylate both wild-type (WT) and familial ALS mutant TDP-43 (M337V TDP-43) under optimized reaction conditions that include magnesium. These conditions support robust phosphorylation of human tau protein, a known substrate of TTBK1/2 (S4A Figure, [31]). Although our preparation of PRKD2 kinase was enzymatically active against a known phosphorylation substrate, histone H1 [34] (S4C Figure), PRKD2 was unable to phosphorylate TDP-43 under any conditions tested, indicating its effect on TDP-43 phosphorylation may be indirect through the activation of other direct TDP-43 kinases or regulation of other downstream members of a TDP-43 regulatory pathway. If the kinases CDC7, TTBK1/2, or PRKD2/3 are in a common regulatory pathway, they may directly phosphorylate one another. Using an *in vitro* kinase assay with purified human kinases, we observed robust auto-phosphorylation by TTBK1 and modest auto-phosphorylation by TTBK2 and PRKD2, consistent with known activities of these kinases [28], [31], [34]. We also tested pairwise combinations of these kinases to determine any relative increases in phosphorylation. However, we did not see any significant increases in phosphorylation on these kinases (S4D Figure). Therefore, any indirect regulation of TDP-43 phosphorylation by PRKD2 may be through other unknown members of one or several regulatory pathways controlling TDP-43 phosphorylation.

### TTBK1/2 promote TDP-43 phosphorylation *in vivo*

**Figure 2:**
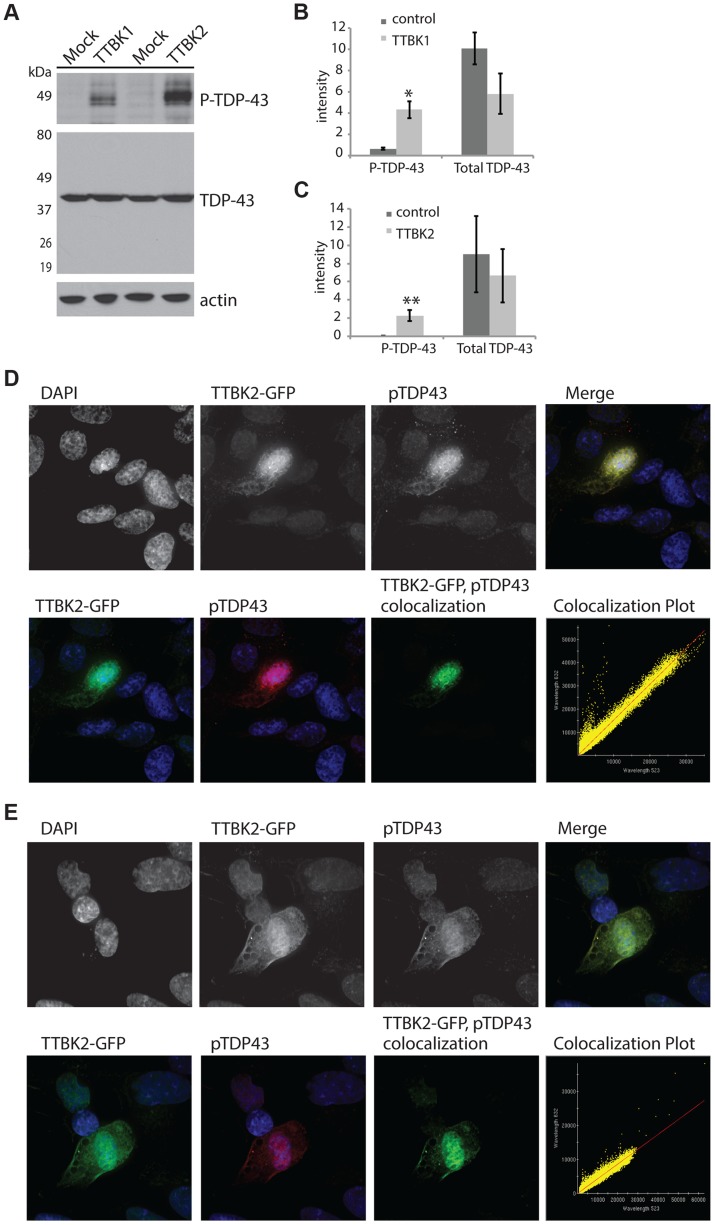
Tau tubulin kinase activation promotes TDP-43 phosphorylation and recruitment into cytoplasmic inclusions. (A) Overexpression of TTBK1 and TTBK2 in HEK293 cells induces robust TDP-43 phosphorylation in the absence of other cellular stressors. Quantitative analysis of band intensities from three independent replicate transfections is shown for (B) TTBK1 and (C) TTBK2. Graphs are plotted in arbitrary units of intensity. *P = 0.004 and **P = 0.035 versus control transfection, Student's t-test. Differences in total TDP-43 are not statistically significant. (D, E) TTBK2 is expressed throughout the cytoplasm, and overlaps with phosphorylated TDP-43 in SHSY-5Y cells. Pearson coefficient of correlation for colocalization (D) = 0.9853, (E) = 0.9793.

TTBK1/2 kinase hyperactivity may contribute to the pathological phosphorylated TDP-43 observed in both FTLD-TDP and ALS. To test whether increased cellular levels of TTBK1/2 activity suffice to drive TDP-43 phosphorylation, we transfected full-length TTBK1 and TTBK2 cDNAs into HEK293 cells. HEK293 cells have some neuronal characteristics and may be derived from a subpopulation of neuronal precursor cells in the embryonic kidney [35]. This cell line is especially useful for biochemical assays requiring high efficiency transfection rates. In the absence of other cellular stresses, we observed robust induction of TDP-43 phosphorylation by immunoblot following transfection with both TTBK1 and TTBK2 (Fig. 2A–C). Likewise, we utilized SH-SY5Y cells, a human neuroblastoma-derived cell line, to determine the location of phosphorylated TDP-43 produced by TTBK2 transfection. The phospho-TDP-43 produced by TTBK2 overexpression is localized throughout the cytoplasm overlapping with TTBK2 (Fig. 2D, E). Further, TTBK2 and phospho-TDP-43 appear concentrated in apparent aggregates, producing a pattern of TDP-43 and TTBK1/2 expression reminiscent of the neuronal cytoplasmic inclusion pathology observed in FTLD-TDP and ALS. SH-SY5Y cells are relatively recalcitrant to transfection; we observed less than 5% transfection efficiency with TTBK2. However, all the cells with strong TTBK2::GFP expression also had inclusions of phosphorylated TDP-43. We observed a similar pattern of TTBK2 transfection overlapping with large phospho-TDP-43 positive aggregates in HEK293 cells (S5 Figure).

**Figure 3:**
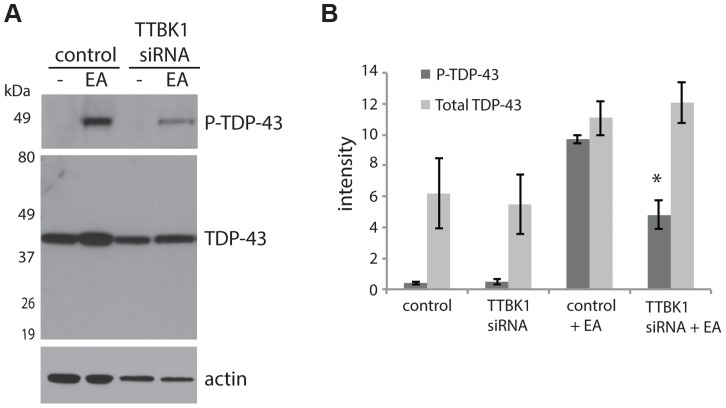
Reduced TTBK1 protects against TDP-43 phosphorylation. (A) NSC-34 cells treated with siRNA targeting TTBK1 exhibit reduced TDP-43 phosphorylation following induction of P-TDP-43 with ethacrynic acid (EA). (B) Quantitative analysis of band intensities from three independent replicate siRNA experiments. Band intensities are graphed in arbitrary units. *P = 0.025 versus control+EA, Student's t-test.

Decreasing TTBK1/2 kinase activity may prevent TDP-43 phosphorylation. To test this hypothesis, we employed small interfering RNAs (siRNAs) to decrease levels of TTBK1 gene expression in mammalian cultured cells. We have modeled pathological TDP-43 phosphorylation in the mouse motor neuron-like NSC-34 cell line using the chemical trigger ethacrynic acid (EA). EA acts by depleting cytosolic and mitochondrial glutathione, resulting in robust TDP-43 phosphorylation [36], [37]. EA is a specific trigger of TDP-43 phosphorylation, because a variety of other cell stressors fail to induce phospho-TDP-43 (S6A Figure). NSC-34 cells were transfected with siRNAs targeting TTBK1, averaging 76% reduction in gene expression and 46% reduction in protein levels (S6B–D Figure). These cells were then treated with EA to induce TDP-43 phosphorylation. We observed a robust decrease in TDP-43 phosphorylation following treatment with TTBK1 siRNA (Fig. 3). We also tested siRNAs targeting TTBK2, but were unable to achieve significant reduction in gene expression.

### TTBK1/2 co-localize with phospho-TDP-43 positive aggregates in FTLD-TDP and ALS

Both TTBK1 and TTBK2 are expressed in the brain, although TTBK2 is expressed in other tissues as well [31], . If TTBK1 or TTBK2 promote TDP-43 phosphorylation in patients with ALS or FTLD, there may be alterations in kinase abundance or localization, and there should be co-occurrence of the kinase with pathological TDP-43 aggregates. Immunohistochemistry for TTBK1, TTBK2 and phospho-TDP-43 was performed on frontal cortex sections from 6 FTLD-TDP cases, 6 ALS cases and 6 normal control cases to determine if there was overlap in the expression of these kinases and their purported target. Additionally, ALS spinal cord and hippocampus were also assessed. One FTLD case carried a progranulin mutation, the remaining 5 are of unknown etiology. FTLD cases were scored according to the harmonized FTLD-TDP classification of pathology [40]. Three of these cases were classified as Type A and three were Type B. All ALS cases were sporadic incidences of disease, and were negative for mutations in TDP-43, SOD1, FUS, and C9ORF72. TTBK1/2 antibody specificity was confirmed against purified substrate, and by antibody competition on fixed tissue (S7 Figure).

**Figure 4:**
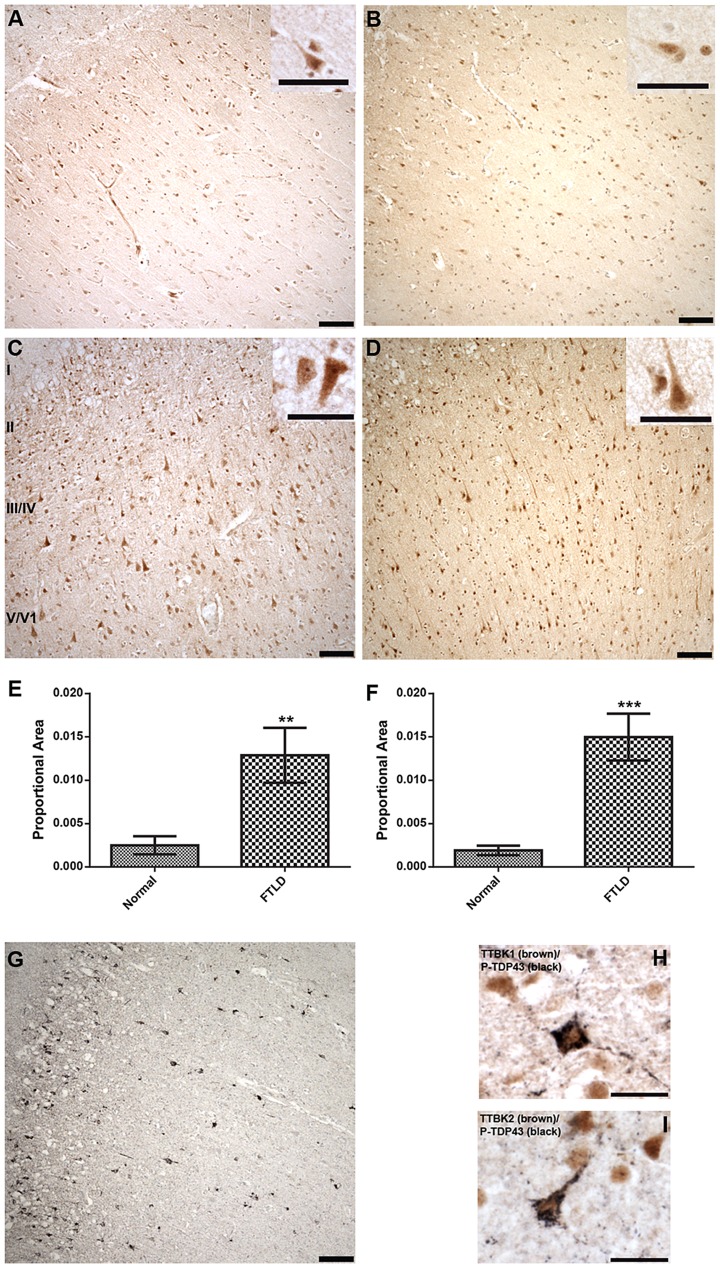
Upregulated Tau tubulin kinases are also co-expressed with phospho-TDP-43 pathology. Representative photomicrographs depicting TTBK1 (A, C) and TTBK2 (B, D) immunoreactivity in cortical neurons in normal (A, B) and FTLD-TDP Type B (C, D) cases. The cellular distribution is both cytoplasmic and nuclear (insets), and immunoreactivity appears to be more widespread in FTLD cases relative to normal controls. Cortical layers I-VI are indicated (C). Quantification of immunostaining demonstrated a statistically significant increase in both TTBK1 (E) and TTBK2 (F) in FTLD cases compared to normal controls (**P = 0.003; ***P<0.0001). The distribution of phospho-TDP-43 immunoreactivity in the cortex of an FTLD case (G) overlaps with TTBK1 (C) and TTBK2 (D). Double label immunohistochemical experiments suggest co-localization of phospho-TDP-43 with TTBK1 (H) and TTBK2 (I) in an FTLD case. Scale bars: 100 µm A–D,G; 50 µm insets A–D; 25 µm H,I. See S5 Figure for controls for antibody specificity.

Fig. 4A–D demonstrates that TTBK1 and TTBK2 immunoreactivity is present in a subset of pyramidal neurons in the frontal cortex of both normal and FTLD cases. Immunoreactivity is more prominent in cortical layers II–VI compared to cortical layer I, where immunoreactivity is relatively sparse, and the cellular localization appears both nuclear and cytoplasmic (Fig. 4, insets). Furthermore, the distribution of TTBK1 and TTBK2 immunoreactivity appears to be more widespread in FTLD cases compared to normal controls. Optical density measurements relative to the proportional area for TTBK1 and TTBK2 immunostaining in frontal cortex confirmed a statistically significant increase in both TTBK1 (Fig. 4E) and TTBK2 (Fig. 4F) immunoreactive distribution in disease-affected subjects. This increase was observed in all FTLD cases surveyed relative to controls. Importantly, the distribution of TTBK1 and TTBK2 in the frontal cortex is consistent with the distribution of phosphorylated TDP-43 pathology in FTLD cases, where aggregates are sparse in cortical layer I, and more abundant in cortical layers II–VI, depending on the FTLD classification (Fig. 4G). To further demonstrate this relationship, we performed double label immunohistochemistry to determine if the tau tubulin kinases and phosphorylated TDP-43 co-expressed within the same neurons. Most neurons immunoreactive for phospho-TDP-43 were also immunoreactive for TTBK1 and TTBK2 (Fig. 4H, I).

**Figure 5:**
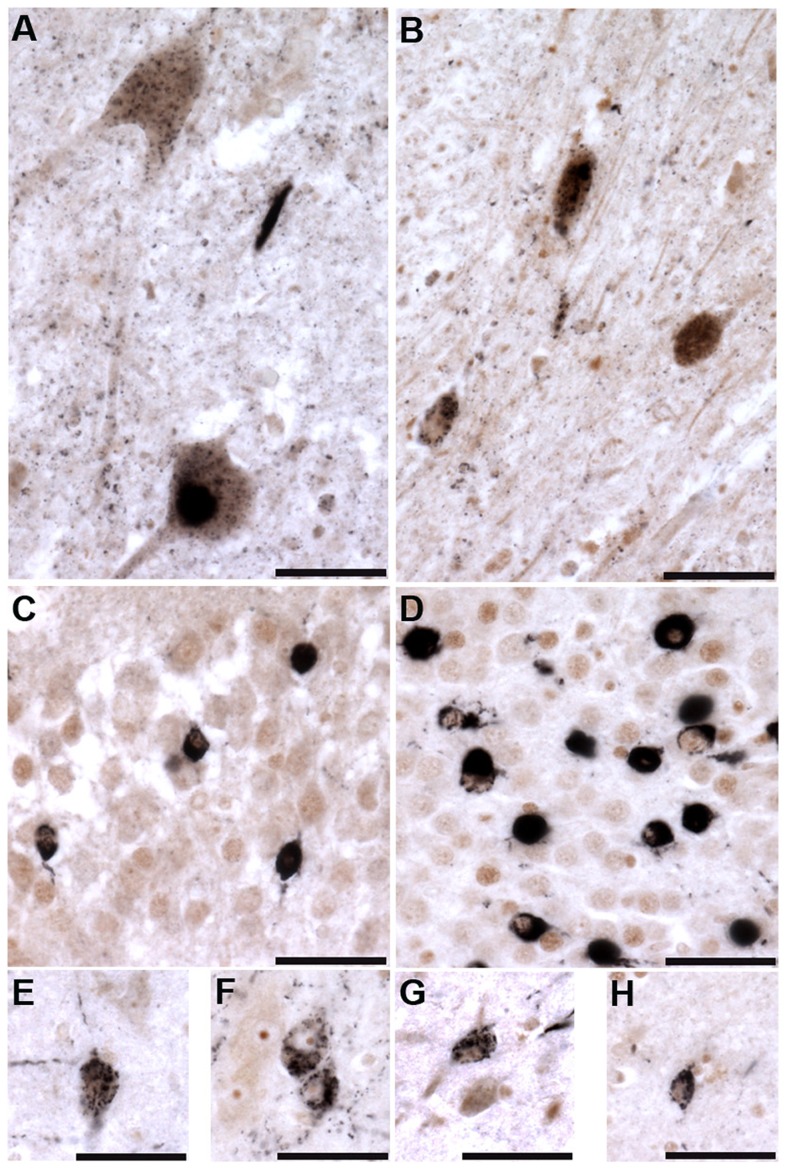
Tau tubulin kinases are co-expressed with phospho-TDP-43 pathology in ALS cases. TTBK1 (brown; A, C, E, F) and TTBK2 (brown; B, D, G, H) co-localize with phospho-TDP-43 (black) in spinal cord motor neurons (A, B), hippocampal dentate granule cells (C, D), cortical neurons (E, G), hippocampal CA3 pyramidal neurons (F) and subiculum (H). Scale bars = 50 µm.

**Figure 6:**
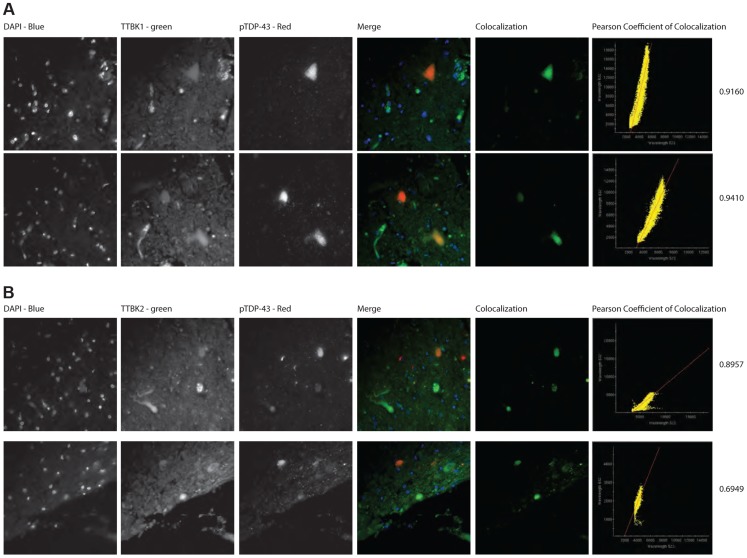
TTBK1/2 co-localize with phosphorylated TDP-43 in ALS spinal cord aggregates. Double-label immunofluorescence of ALS spinal cord of (A) TTBK1 and (B) TTBK2 show significant co-localization of TTBK1/2 with phospho-TDP-43 within neuronal cytoplasmic inclusions. Significance was determined using Pearson coefficient of colocalization.

Of the six ALS cases examined, only two had phospho-TDP-43 aggregates in the frontal cortex and hippocampus, while all six demonstrated phospho-TDP-43 aggregates within spinal cord. ALS spinal cord motor neurons immunoreactive for phospho-TDP-43 pathology also co-labeled with TTBK1 and TTBK2 (Fig. 5A, B). Of the two ALS cases with pathologic changes in brain, a subset of neurons in the hippocampus and frontal cortex containing phospho-TDP-43 aggregates also co-expressed TTBK1 and TTBK2, while other neurons appeared to be immunoreactive for phospho-TDP-43 alone (Fig. 5 C–H). To test whether TTBK1/2 co-localize with phosphorylated TDP-43, we performed double-label immunofluorescence on ALS spinal cord sections (Fig. 6 and S8 Figure). In general, more neurons were immunofluorescent for TTBK1/2 than for phosphorylated TDP-43. Similar to our double label immunohistochemical data, neurons immunofluorescent for phosphorylated TDP-43 usually co-localized with TTBK1/2, although some neurons labeled with phosphorylated TDP-43 alone. Taken together Figs. 4, 5 and 6 repeatedly demonstrate an overlapping expression pattern for TTBK1/2 and pS409/410 TDP-43 inclusions in ALS and FTLD-TDP consistent with TTBK1/2 participation in the genesis of TDP-43 lesions.

## Discussion

Tandem phosphorylation at TDP-43 serines 409 and 410 (pS409/410) is a consistent and robust feature of TDP-43 pathology in ALS and FTLD-TDP. Our previous work in TDP-43 transgenic *C. elegans* demonstrated a causal relationship between neurodegeneration and S409/410 phosphorylation of TDP-43 [14], [23]. We have utilized this model as a *C. elegans* behavior-based screening tool to identify TDP-43 kinases [23]. However, it is possible other relevant TDP-43 kinases remain unidentified. To uncover kinases responsible for the pathological phosphorylation of TDP-43, we have re-screened the *C. elegans* kinome by RNAi knockdown for modifiers of TDP-43 phosphorylation. For this survey we employed sensitive and specific S409/410 phosphorylation dependent TDP-43 antibodies [41] to directly detect changes in TDP-43 phosphorylation state following RNAi treatment. Confirmation of identified candidate kinases in *C. elegans* was conducted by testing deletion mutations within the kinase genes of interest. Three identified candidate kinases, *cdc-7*, *dkf-2*, and H05L14.1, reduced TDP-43 S409/410 phosphorylation and improved TDP-43 dependent behavioral phenotypes in *C. elegans*. TDP-43 is a known substrate of CDC7, as it was previously uncovered in a reverse genetic screen to identify modifiers of TDP-43 behavioral phenotypes [23], confirming the validity of this approach. We employed standard BLAST protein sequence homology searching algorithms [42] to identify the closest mammalian homologs of our novel TDP-43 kinases *dkf-2* and H05L14.1. Interestingly, H05L14.1 was a homolog of the mammalian tau tubulin kinases TTBK1 and TTBK2. Our previous behavior-based screen for TDP-43 kinases identified a different *C. elegans* homolog of TTBK1/2 as a TDP-43 kinase, although at the time we were unable to demonstrate a direct relationship between human TTBK1/2 and TDP-43 [23]. We decided to re-evaluate TTBK1/2 as we had identified both as candidate kinases in independent assays. Using optimized *in vitro* kinase reaction conditions, we demonstrate here that human TTBK1/2 are able to directly phosphorylate TDP-43. We then overexpressed TTBK1/2 in cultured human cells. TTBK1/2 overexpression in the absence of other stressors promoted robust phosphorylation of endogenous TDP-43. Furthermore, this phosphorylated TDP-43 localized to the cytoplasm in inclusion-like aggregates. We also found that reduction of TTBK1 mRNA levels attenuated TDP-43 phosphorylation in a chemically induced model of pathological phospho-TDP-43 accumulation. Finally, to explore whether TDP-43 kinase hyperactivity could underlie the etiology of TDP-43 proteinopathies, we immunostained tissue from FTLD-TDP and ALS for TTBK1/2. We observe increased TTBK1/2 in FTLD-TDP frontal cortex, and co-localization with TDP-43 positive aggregates in FTLD frontal cortex and ALS spinal cord. One possible explanation for these data is the observed differences in TTBK1/2 expression drives neurodegeneration in TDP-43 proteinopathies. Taken together these data support a pivotal role for TTBK1/2 hyperactivity in TDP-43 proteinopathy.

A number of kinases have been identified to date with the ability to phosphorylate TDP-43 *in vitro* and *in vivo*. The kinases CK1 [43], [44], CKII [45], CDC7 ([23], this study), TTBK1 and TTBK2 (this study) may all contribute to pathological TDP-43 phosphorylation in humans; regardless they all share target sequence conservation as the CK1 kinase domain is the prototypical model for this family of kinases. CK1 family kinases may act redundantly to regulate TDP-43 phosphorylation in a common signaling pathway. Alternatively, extracellular and intracellular signals may act as a trigger to specify kinase activity from one of the available TDP-43 kinases but not the others. We have observed that in the absence of each of these known TDP-43 kinases in *C. elegans*, mutant TDP-43 still exhibits varying but detectable degrees of phosphorylation (Fig. 1 and [23]), indicating that no one kinase accounts for all observed TDP-43 phosphorylation. Exploring the functional relationships between and regulatory networks governing the TDP-43 kinases identified to date will be important future work.

TTBK1 and TTBK2 were originally purified on the basis of their kinase activity on the microtubule binding protein tau at several pathological phospho-tau epitopes known to accumulate in Alzheimer's disease [31], [38], [39], [46]. Tangles composed of insoluble hyperphosphorylated tau are a pathological hallmark of Alzheimer's disease (AD), as well as a number of other neurodegenerative diseases including FTLD-tau, progressive supranuclear palsy (PSP), and chronic traumatic encephalopathy (CTE). Interestingly, phosphorylated TDP-43 is also present in a subset of patients with primary tauopathies such as AD, PSP, and CTE [8], [47], [48], and either tau or TDP-43 are the diagnostic pathologic changes in the vast majority of frontotemporal lobar degeneration cases [49]. The relationship between TDP-43 and tau neuropathologic changes remains unclear. One hypothesis is that both proteinopathy disorders share a common etiology in TTBK1/2 activation leading to either TDP-43 or tau neuropathology depending on the vulnerable cell population affected by TTBK activation. The downstream toxic mechanisms for tau and TDP-43 appear distinct; however, inappropriate TTBK1 and TTBK2 activity may constitute a shared mechanistic link in initiating both tau and TDP-43 neuropathologies.

Both TTBK1 and TTBK2 have been previously implicated in neurodegenerative diseases. Single nucleotide polymorphisms (SNPs) in TTBK1 are associated with decreased Alzheimer's disease risk in studies of Spanish and Han Chinese populations [50], [51]. TTBK1 has been shown to co-localize with diffuse phospho-Ser422 tau in pre-tangle Alzheimer's disease neurons [52], and increased levels of TTBK1 have been observed in AD frontal cortex [53] and enhance the toxicity of tau in a P301L mouse model [54]. Mutations in TTBK2 have been shown to cause spinocerebellar ataxia type 11 (SCA11) [55], a progressive neurodegenerative disorder characterized by tau pathology. Mouse models heterozygous for mutant TTBK2 exhibit decreased TTBK2 kinase activity and altered TTBK2 localization, while homozygous mutant TTBK2 is embryonic lethal [56]. Our results are the first demonstration of a potential role for TTBK1 and TTBK2 in primary TDP-43 proteinopathies.

Kinases regulating TDP-43 phosphorylation present an attractive target for therapeutic intervention in both ALS and FTLD-TDP. No specific small molecule inhibitors targeting TTBK1 or TTBK2 has been reported to date, despite their potential roles contributing to tauopathies by hyperphosphorylating tau. Development of brain penetrant TTBK1 and TTBK2 inhibitors may also provide a viable strategy for intervening in TDP-43 proteinopathy disorders including ALS and FTLD-TDP.

## Materials and Methods

### Transgenics and strains

N2 (Bristol) was used as wild type *C. elegans* and maintained as previously described [57]. Strains were maintained at 16°C. Experiments involving *C. elegans* were performed at room temperature unless otherwise noted. CK423 (TDP-43 M337V) and *eri-1(mg366);lin-15b(n744)*;TDP-43 M337V transgenic strains were generated previously [14], [23]. Kinase mutants were crossed with CK423 to generate strains CK566 *cdc-7(tm4391)*;TDP-43, CK602 H05L14.1(tm4720);TDP-43, CK600 *dkf-2(tm4076)*;TDP-43, CK597 *nsy-1(ok593)*;TDP-43, CK613 *kin-20(ok505)*;TDP-43, CK574 *mlk-1(ok2471)*;TDP-43, CK623 F39F10.3(tm4396);TDP-43.

### RNAi screen

The list of predicted kinase genes in *C. elegans* was derived from the *C. elegans* kinome project [58], with library construction as described [23]. Testing was done in an *eri-1(−/−);lin-15(−/−)* RNAi enhancing mutant background [59]. Staged embryos were plated, grown at 16°C for 8–9 days, and then a mixed population of 1^st^ generation gravid adults with 2^nd^ generation L2–L3 animals were harvested by washing with M9 buffer into 96 well plates and frozen at −80°C, for subsequent immunoblot analysis. Each RNAi treated population was evaluated semi-quantitatively for reduction in phospho-TDP-43 relative to control treated animals. Positives candidates were retested for effects on TDP-43 phosphorylation by independent RNAi treatment and immunoblot, and the RNAi gene target for each plasmid was confirmed by sequencing.

### Immunoblotting

Equivalent mixed-stage worm lysate fractions were loaded and resolved on precast 4–15% gradient SDS-PAGE gels and transferred to PVDF membrane as recommended by the manufacturer (Bio-Rad). On immunoblots, human TDP-43 was detected with a commercially available monoclonal antibody ab57105 (Abcam) directed against human TDP-43 amino acids 1–261. TDP-43 phosphorylated at S409/S410 was detected by a monoclonal antibody called anti phospho TDP-43 (pS409/410) available from Cosmobio (catalog # TIP-PTD-M01). *C. elegans* β-tubulin levels were measured using monoclonal antibody E7 as a loading control as previously described [60], [61]. TTBK1 was detected by Abcam rabbit polyclonal antibody ab103944 at 1∶1000 dilution. TTBK2 was detected by Abgent rabbit polyclonal antibody AP12162a at 1∶1000 dilution. HRP labeled goat anti-mouse IgG was the secondary antibody (GE Healthcare) and used at a dilution of 1∶4000. Dilutions were: 1∶7500 for ab57105, 1∶1000 for pS409/410, and 1∶10000 for E7. Immunoblots shown are representative of at least 3 independent experiments. Quantitation was performed using ImageJ image processing and analysis software.

### Kinase assays

GST-TDP-43 (WT) and GST-TDP-43 (M337V) fusion proteins were purified from BL21 (DE3) expression host cells as previously described [62]. Active kinase enzymes were obtained commercially via purification from SF9 cells for PRKD2, TTBK1 and TTBK2 (Signalchem). Enzyme assays were carried out in a kinase reaction buffer containing 25 mM MOPS, 12.25 mM glycerol-phosphate, 25 mM MgCl, 5 mM EGTA, 2 mM EDTA, 0.25 mM DTT and 50 µM ATP.

### Cell lines

HEK 293 cells (ATCC) were cultured in Dulbecco's modified Eagle medium (DMEM) supplemented with 10% defined fetal bovine serum (FBS) and penicillin (50 IU/ml)–streptomycin (50 mg/ml). NSC-34 cells (Cedarlane Labs) and SHSY-5Y cells (ATCC) were cultured in DMEM/HAM's F12 (50/50) with 10% FBS and penicillin (50 IU/ml)–streptomycin (50 mg/ml).

### Immunofluorescence for cultured cells

Cells were seeded onto poly-D-Lysine coated (Sigma Aldrich) 12 mm round glass cover slips in 24-well plates. Cells were transfected with the plasmid encoding TTBK2-GFP with GenePorter 2 (Genlantis) using the manufacturer's protocol. Cells were fixed for imaging in 4% formaldehyde 96 hours after transfection. Cells were washed 3×5 min in PBS/Ca^2+^/Mg^2+^, then blocked in antibody buffer (PBS, 0.5% Triton X-100, 1 mM EDTA, 0.1% BSA, 0.05% NaN_2_)+10% normal goat serum. Primary antibody was applied and incubated for 1 hour at room temperature (Cosmo Bio; 1∶1000). Cells were washed 3×5 min in PBS/Ca^2+^/Mg^2+^, then re-blocked for 10 min. Appropriate secondary antibody was applied and incubated for 20 min at room temperature. Cells were again washed 3×5 min in PBS/Ca^2+^/Mg^2+^, counterstained with 300 nM DAPI and mounted with ProLong Gold antifade. Microscopy was performed on a Delta Vision microscope (Applied Precision, Inc) using a 60× oil immersion objective, a sCMOS camera, and 2×2 binning. Image analysis was performed using softWoRx 6.0 Beta software.

### RNA interference

HEK 293 cells were treated with 150 µM ethacrynic acid (EA) for 5 hours to induce endogenous TDP-43 phosphorylation [36]. NSC-34 cells were grown in differentiation medium (DMEM/HAM's F12 (50/50), 1% FBS, 1% non-essential amino acids (NEAA), penicillin (50 IU/ml)–streptomycin (50 mg/ml)) for one day prior to treatment with 50 µM EA for 5 hours. TTBK1 siRNA construct was MMC.RNAI.N001162864.12.1 (Integrated DNA Technologies). RNAi experiments were carried out as per protocol in the TriFECTa Dicer-Substrate RNAi manual (Integrated DNA Technologies).

### Transfection

Transfection of plasmids containing full-length TTBK1 (pWO:TTBK1) and TTBK2 (TTBK2 GFP pFLAP dest) sequences [63] was performed as specified by the manufacturer using the Geneporter 2 Transfection Reagent (Genlantis).

### Quantitative reverse-transcription PCR

RNA was purified from flash-frozen cell pellets using TRIzol Reagent (Life Technologies) according to the manufacturer's protocol. cDNA was made using iScript Reverse Transcription Supermix (Bio-Rad). qPCR was performed on an 7900HT Real Time PCR System (Applied Biosystems) using iTaq Universal SYBR Green Supermix (Bio-Rad).

### Ethics statement: Post mortem human tissue

De-identified post-mortem brain tissue used in this study was determined to be an exempt from IRB review by the VA Puget Sound Health Care System Human Research Protection Program Director on December 29, 2011. Tissue used for these studies was obtained from the University of Washington Alzheimer's Disease Research Center brain bank (Seattle, WA), and the Indiana Alzheimer Disease Center brain bank(Indianapolis, IN), where consent for autopsy and permission for use of tissue in scientific experiments was obtained. FTLD and ALS cases were selected on the basis of having an autopsy-confirmed diagnosis of FTLD and FTLD-related disorders or ALS. Control samples were from de-identified neurologically healthy control participants, who were of a similar age.

### Immunohistochemistry and immunofluorescence for tissue

Primary antibodies used for immunohistochemistry were anti-TTBK1 (Abcam, 1∶100), anti-TTBK2 (Abgent, 1∶200), and anti-phospho TDP-43 409/410 (CosmoBio, 1∶1000)). In order to minimize variability, sections from all cases (normal and affected subjects) were stained simultaneously for each antibody. Immunostained sections were analyzed using the computerized image analysis system, MicroComputer Imaging Device (MCID, Imaging Research, St. Catherines, Ontario, Canada). Blinded assessment of optical density measurements were obtained relative to the proportional area for TTBK1 and TTBK2 immunostaining in frontal cortex grey matter (three separate readings per case). Data were averaged and are represented as mean +/− SEM. A two tailed Student's t-test was used to assess differences in TTBK1 and TTBK2 expression between cases and controls. For double label immunohistochemistry experiments, sections were first immunostained with anti-phospho TDP-43 and reaction product was visualized with nickel enhanced DAB (black). Sections were then immunostained with anti-TTBK1 or TTBK2 and visualized with DAB alone (brown). For double label immunofluorescence experiments, AlexaFluor 488 goat anti-rabbit and AlexaFluor 594 goat anti-mouse secondary antibodies (Molecular Probes) were used and autofluoresence was quenched with 0.1% Sudan Black [64]. To demonstrate specificity of the TTBK antibodies, TTBK1 and TTBK2 were blocked with 50-fold amount of immunizing peptide overnight at 4°C before proceeding with the immunostaining protocol (see S5 Figure).

## Supporting Information

S1 Figure Immunoblot results from primary kinase RNAi screen. Populations of RNAi treated *C. elegans* were harvested into 96-well plates prior to immunoblot analysis. Gene names and locations of kinases tested are presented in **Table S1**. Two rows from each plate were tested in alternating wells for each immunoblot. Labels above individual wells describe Row and Column information for each sample. Plate numbers are indicated at the left of the immunoblot. α-tubulin antibody is used as a load control. Candidates confirmed on repeat testing are boxed in blue.
(PDF)

S2 Figure Full immunoblots of total TDP-43 levels. Full immunoblots from Fig. 1, showing low (3 minute exposure) and high molecular weight species (15 minute exposure) of total TDP-43.
(PDF)

S3 Figure H05L14.1 and *dkf-2* mammalian homologs identified by BLAST. (A, B) Cladogram of vertebrate homologs of the *C. elegans* proteins H05L14.1 and *dkf-2*. The entire *C. elegans* amino acid sequences of H05L14.1 or *dkf-2* were compared against non-redundant reference sequences from the RefSeq protein database (7-20-2014, NCBI). (A) 2878 active hits were identified by BLAST with similarity to H05L14.1, and subsequently filtered to include only sequences from *C. elegans* or phylum chordata. The top 50 hits underwent multiple sequence alignment, alignment refinement, phylogenetic reconstruction, and are displayed in a cladogram, with branch support values in red [27]. Related human gene and gene identifier is boxed. Homo sapiens GI# 58761548 is TTBK1. (B) 5000 active hits were identified with similarity to *dkf-2*, filtered, and graphed as above. Homo sapiens GI# 5031689 is PRKD3. (C) H05L14.1 kinase domain has 40% identity to human TTBK1 and TTBK2. (D) *dkf-2* has more than 70% identity to human PRKD2 and PRKD3. Sequence identity was calculated using Clustal W method for multiple sequence alignment. (E) Alignment report for H05L14.1, TTBK1, and TTBK2 kinase domain, including boxes around sequence that matches the consensus. (F) Alignment report for *dkf-2*, PRKD2, and PRKD3. Reports were generated using Lasergene MegAlign software for protein sequence analysis and alignment.
(PDF)

S4 Figure *In vitro* kinase assay controls. (A) Tau is a known substrate of TTBK2 [65]. To test enzyme activity, approximately 1 µg of non-phosphorylated recombinant human tau purified from *E. coli* were incubated with equivalent amounts of TTBK2 enzyme purified from cultured cells by two commercial suppliers (Origene catalog #LY406582 and Signalchem #T18-11G). Phosphorylation was assessed by reactivity with AT270, a phospho-tau antibody recognizing tau phosphorylated at Thr181. (B) Purified TTBK1, TTBK2, and CDC7 can also phosphorylate TDP-43 at serines 403 and 404 (CosmoBio, #CAC-TIP-PTD-P05) in an *in vitro* kinase assay. (C) Histone H1 is a known substrate of PRKD2 [34]. To confirm PRKD2 activity, human PRKD2 (SignalChem #P76-10) purified from cultured cells was incubated with purified recombinant Histone H1. We observed phosphorylation of Histone H1 as detected by reactivity with pT146 specific antibody (Bioss Catalog # bs-3176R). (D) Purified CDC7, TTBK1, TTBK2, or PRKD2 were incubated singly or pairwise with radiolabeled phosphate. TTBK1 can robustly auto-phosphorylate, while TTBK2 and PRKD2 are also capable of auto-phosphorylation. Pairwise combinations of CDC7, TTBK1, TTBK2, and PRKD2 did not exhibit any increase or variety in phosphorylation beyond baseline levels of auto-phosphorylation for each kinase.
(PDF)

S5 Figure (A) Phosphorylated TDP-43 is localized in a large discrete cytoplasmic aggregate following TTBK2 overexpression in HEK293 cells. (B) TTBK2 and phosphorylated TDP-43 co-localize in cells overexpressing TTBK2.
(PDF)

S6 Figure (A) Treatment of HEK293 cells with a variety of cellular stressors failed to produce phosphorylated TDP-43. Bafilomycin and wortmannin are inhibitors of autophagy, PSI is a general proteasome inhibitor, cadmium chloride is a heavy metal, taxol is an inhibitor of microtubule dynamics, rotenone blocks the mitochondrial electron transport chain (creating reactive oxygen species (ROS)), pepstatin inhibits aspartic proteases, and paraquat catalyzes formation of ROS. (B) TTBK1 is reduced by nearly 80% following siRNA treatment in NSC-34 cells. Quantitative PCR measurements (qPCR) for TTBK1 mRNA levels are displayed in arbitrary units for an untreated control and cells treated with TTBK1 siRNA. (C) siRNA targeting TTBK1 reduce levels of TTBK1 protein, as detected by immunoblot. (D) TTBK1 protein levels are reduced by an average of 46%, following siRNA treatment in NSC-34 cells. Quantitation of TTBK1 protein levels from three independent experiments is graphed in arbitrary units of band intensity.
(PDF)

S7 Figure Antibody Validation. (A) Studies examining expression of TTBK1 used commercially sourced antibodies including Anti-TTBK1 (Sigma-Aldrich, SAB3500002), Anti TTBK#1 (Abgent, AP4947a), Anti-TTBK1 (**Abcam, ab103944**). (B) Antibodies tested for TTBK2 were TTBK2 Antibody N-term (**Abgent, AP12162a**), Anti-Tau tubulin kinase 1 antibody (Abcam, ab67839), and TTBK2 Polyclonal Antibody (Proteintech, 15072-1-AP). Antibodies underlined/bold above were used in further experiments and are boxed in red in the figure. (C–J) Peptide blocking experiments with the cognate immunizing peptide further demonstrates specificity of the selected TTBK1 and TTBK2 antibodies. Anti-TTBK1 (**Abcam, ab103944**) (C–F) and anti-TTBK2 (**Abgent, AP12162a**) (G–J) were pre-incubated with a 50 fold excess of the blocking peptide (D, F, H, J) before proceeding with the immunostaining protocol and compared with immunostaining using antibody alone (C, E, G, I). ALS spinal cord (C, D, G, H) and FTLD frontal cortex (E, F, I, J). Scale bar = 100 um.
(PDF)

S8 Fig TTBK1/2 co-localize with phosphorylated TDP-43 in aggregates in ALS spinal cord. Double-label immunofluorescence of ALS spinal cord demonstrates additional neurons that significantly co-localize TTBK1 (upper panel) or TTBK2 (lower panel) with phospho-TDP-43 within neuronal cytoplasmic inclusions. Significance was determined using Pearson coefficient of colocalization.
(PDF)

S1 Table Kinase genes tested by immunoblot. 96-well plate locations of each RNAi treated population of *C. elegans* prior to testing by immunoblot (S1 Figure). Control RNAi for each plate are highlighted in purple. L4440: empty vector RNAi control. *unc-22*: positive control for effective RNAi treatments, causing a strong paralyzed phenotype in treated TDP-43 worms. TDP-43: RNAi targeting the TDP-43 transgene is a positive control for suppression of TDP-43 phenotypes. Kinase RNAi treatments that caused *C. elegans* sterility or growth arrest, causing insufficient sample for protein detection by immunoblot, are highlighted in green. Kinase RNAi treatments that decreased TDP-43 phosphorylation in initial testing are highlighted in blue. Kinase RNAi treatments that reproducibly decreased TDP-43 phosphorylation in multiple independent experiments have names that are bolded and underlined.
(XLSX)
