# Supplementary figures and images for: The Tau Tubulin Kinases TTBK1/2 Promote Accumulation of Pathological TDP-43
Source: PLoS Genet. 2014 Dec 4;10(12):e1004803. doi: 10.1371/journal.pgen.1004803 (PMC4256087; doi:10.1371/journal.pgen.1004803)

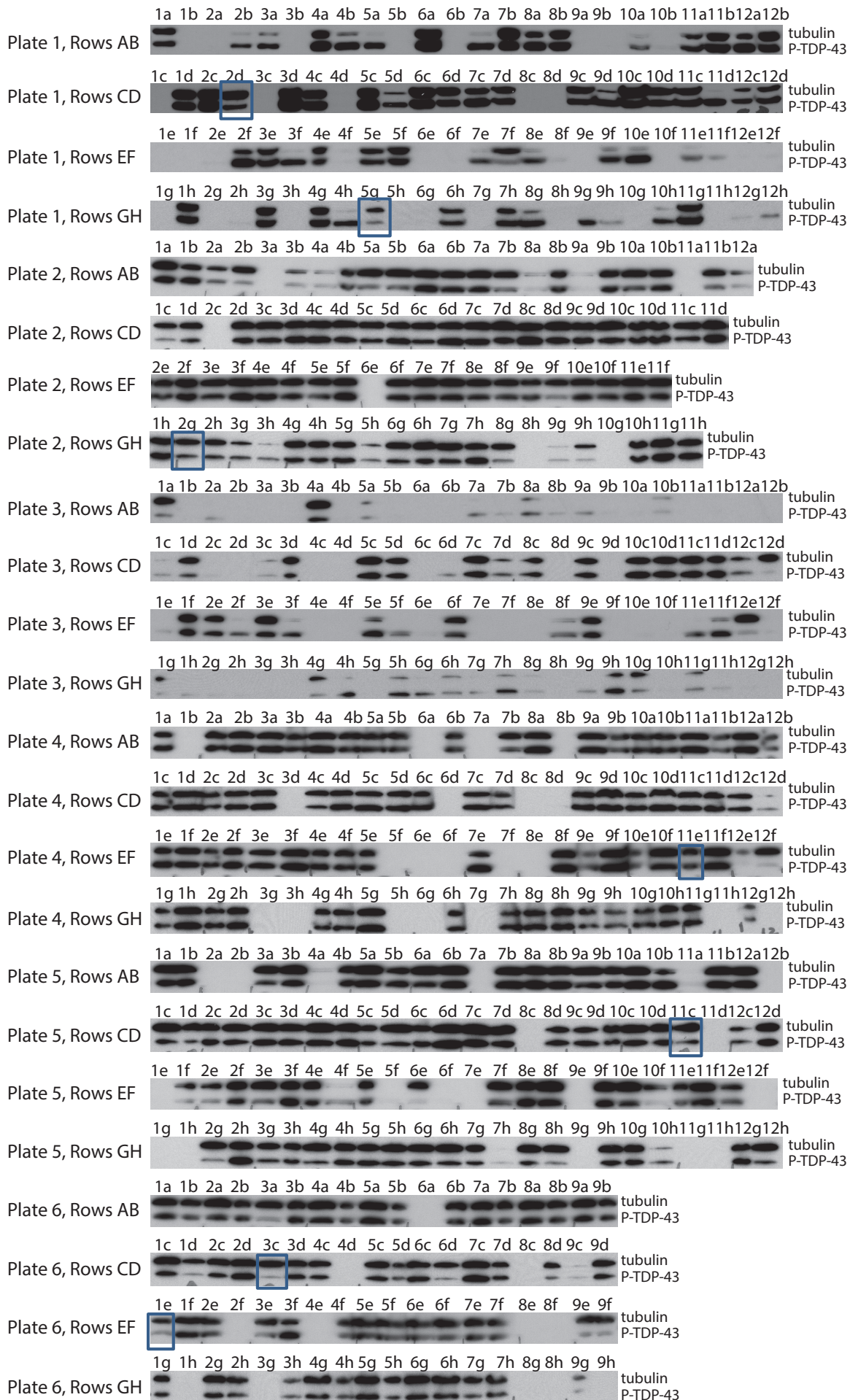

Supplement: S1 Figure — Immunoblot results from primary kinase RNAi screen. Populations of RNAi treated C. elegans were harvested into 96-well plates prior to immunoblot analysis. Gene names and locations of kinases tested are presented in Table S1. Two rows from each plate were tested in alternating wells for each immunoblot. Labels above individual wells describe Row and Column information for each sample. Plate numbers are indicated at the left of the immunoblot. α-tubulin antibody is used as a load control. Candidates confirmed on repeat testing are boxed in blue. (PDF) [file pgen.1004803.s001.pdf]

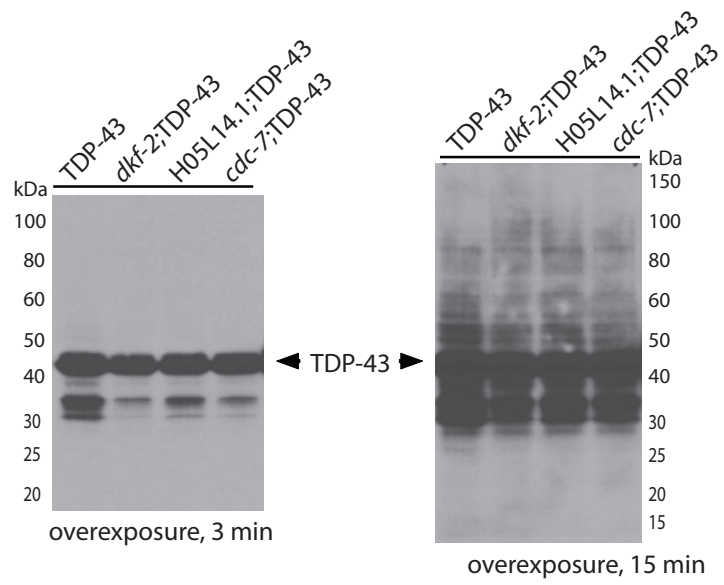

Supplement: S2 Figure — Full immunoblots of total TDP-43 levels. Full immunoblots from Fig. 1, showing low (3 minute exposure) and high molecular weight species (15 minute exposure) of total TDP-43. (PDF) [file pgen.1004803.s002.pdf]

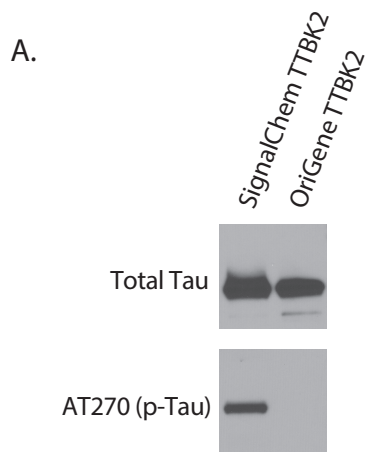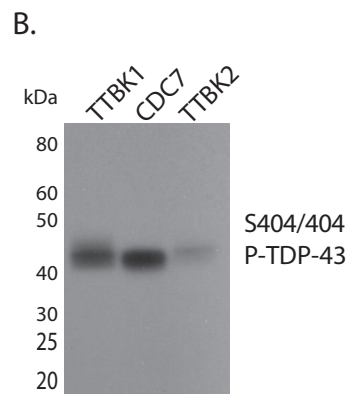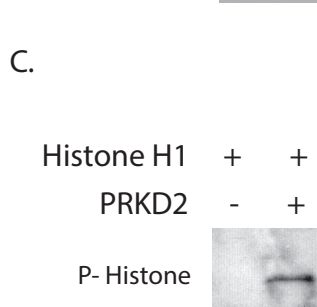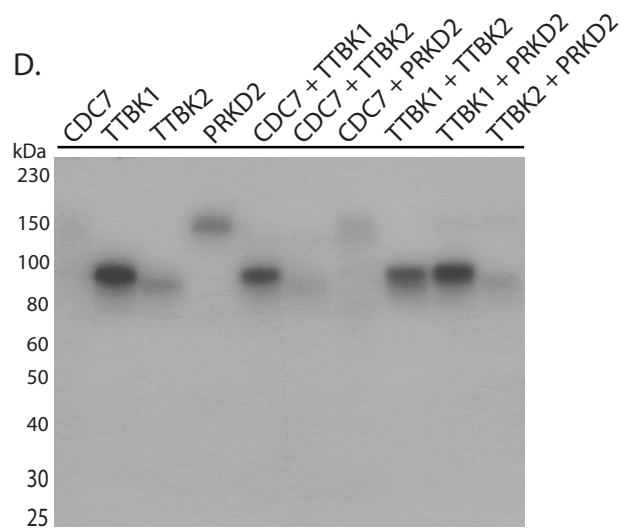

Supplement: S4 Figure — In vitro kinase assay controls. (A) Tau is a known substrate of TTBK2 [65]. To test enzyme activity, approximately 1 µg of non-phosphorylated recombinant human tau purified from E. coli were incubated with equivalent amounts of TTBK2 enzyme purified from cultured cells by two commercial suppliers (Origene catalog #LY406582 and Signalchem #T18-11G). Phosphorylation was assessed by reactivity with AT270, a phospho-tau antibody recognizing tau phosphorylated at Thr181. (B) Purified TTBK1, TTBK2, and CDC7 can also phosphorylate TDP-43 at serines 403 and 404 (CosmoBio, #CAC-TIP-PTD-P05) in an in vitro kinase assay. (C) Histone H1 is a known substrate of PRKD2 [34]. To confirm PRKD2 activity, human PRKD2 (SignalChem #P76-10) purified from cultured cells was incubated with purified recombinant Histone H1. We observed phosphorylation of Histone H1 as detected by reactivity with pT146 specific antibody (Bioss Catalog # bs-3176R). (D) Purified CDC7, TTBK1, TTBK2, or PRKD2 were incubated singly or pairwise with radiolabeled phosphate. TTBK1 can robustly auto-phosphorylate, while TTBK2 and PRKD2 are also capable of auto-phosphorylation. Pairwise combinations of CDC7, TTBK1, TTBK2, and PRKD2 did not exhibit any increase or variety in phosphorylation beyond baseline levels of auto-phosphorylation for each kinase. (PDF) [file pgen.1004803.s004.pdf]

A.

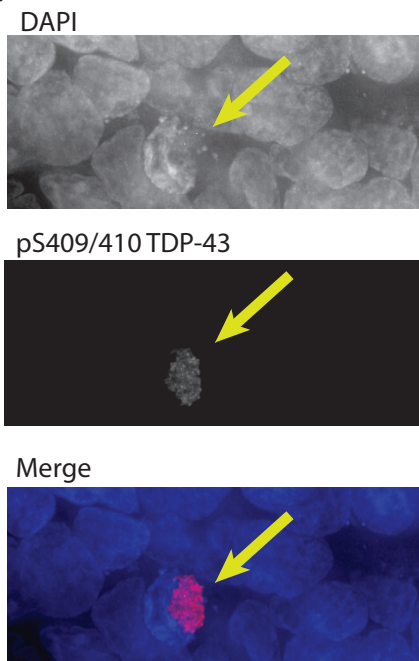

B.

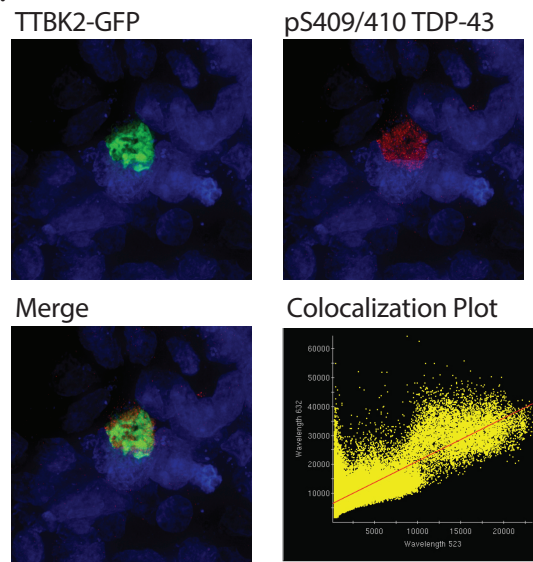

Supplement: S5 Figure — (A) Phosphorylated TDP-43 is localized in a large discrete cytoplasmic aggregate following TTBK2 overexpression in HEK293 cells. (B) TTBK2 and phosphorylated TDP-43 co-localize in cells overexpressing TTBK2. (PDF) [file pgen.1004803.s005.pdf]

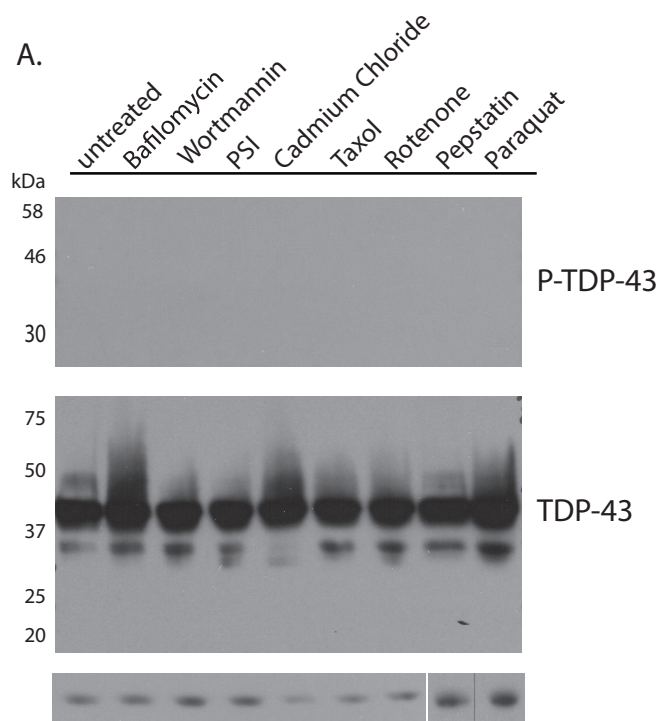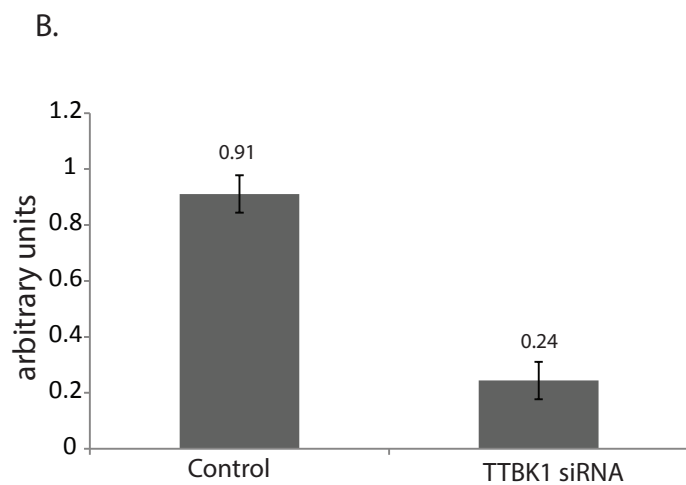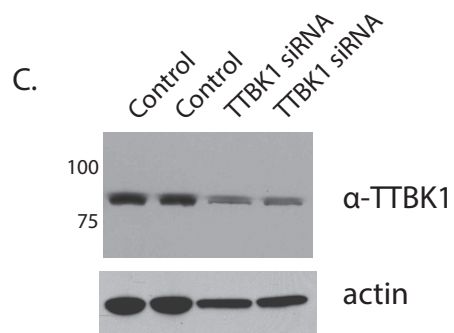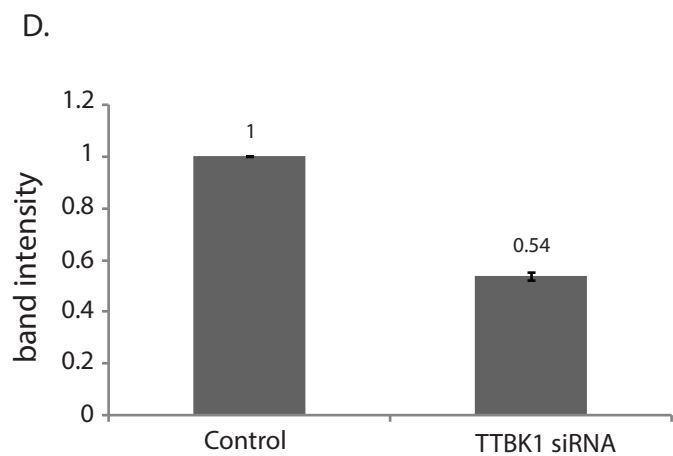

Supplement: S6 Figure — (A) Treatment of HEK293 cells with a variety of cellular stressors failed to produce phosphorylated TDP-43. Bafilomycin and wortmannin are inhibitors of autophagy, PSI is a general proteasome inhibitor, cadmium chloride is a heavy metal, taxol is an inhibitor of microtubule dynamics, rotenone blocks the mitochondrial electron transport chain (creating reactive oxygen species (ROS)), pepstatin inhibits aspartic proteases, and paraquat catalyzes formation of ROS. (B) TTBK1 is reduced by nearly 80% following siRNA treatment in NSC-34 cells. Quantitative PCR measurements (qPCR) for TTBK1 mRNA levels are displayed in arbitrary units for an untreated control and cells treated with TTBK1 siRNA. (C) siRNA targeting TTBK1 reduce levels of TTBK1 protein, as detected by immunoblot. (D) TTBK1 protein levels are reduced by an average of 46%, following siRNA treatment in NSC-34 cells. Quantitation of TTBK1 protein levels from three independent experiments is graphed in arbitrary units of band intensity. (PDF) [file pgen.1004803.s006.pdf]

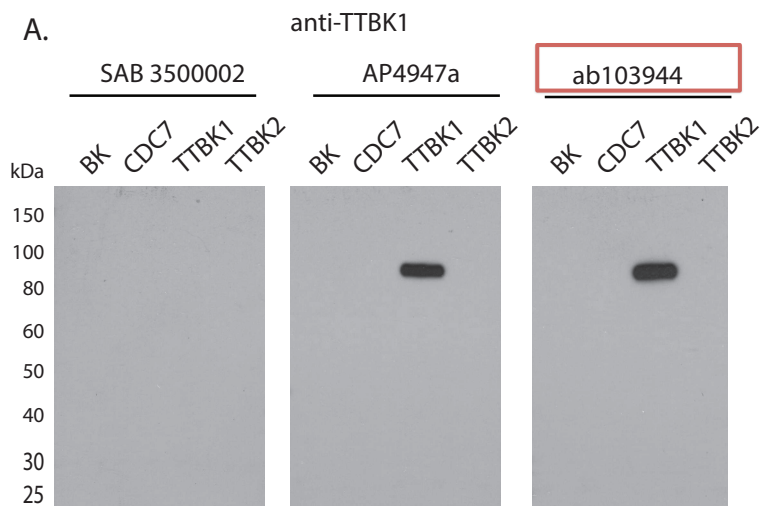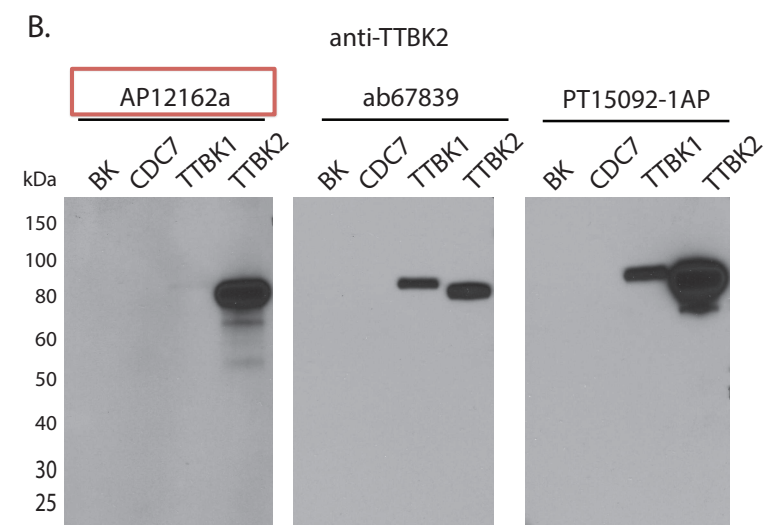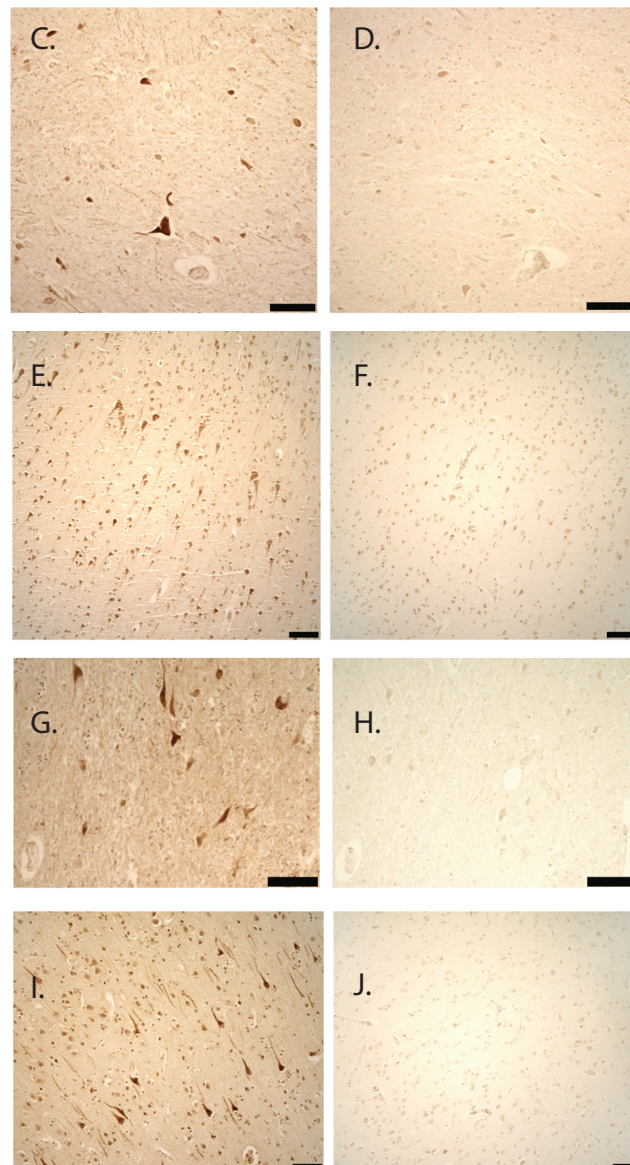

Supplement: S7 Figure — Antibody Validation. (A) Studies examining expression of TTBK1 used commercially sourced antibodies including Anti-TTBK1 (Sigma-Aldrich, SAB3500002), Anti TTBK#1 (Abgent, AP4947a), Anti-TTBK1 (Abcam, ab103944). (B) Antibodies tested for TTBK2 were TTBK2 Antibody N-term (Abgent, AP12162a), Anti-Tau tubulin kinase 1 antibody (Abcam, ab67839), and TTBK2 Polyclonal Antibody (Proteintech, 15072-1-AP). Antibodies underlined/bold above were used in further experiments and are boxed in red in the figure. (C–J) Peptide blocking experiments with the cognate immunizing peptide further demonstrates specificity of the selected TTBK1 and TTBK2 antibodies. Anti-TTBK1 (Abcam, ab103944) (C–F) and anti-TTBK2 (Abgent, AP12162a) (G–J) were pre-incubated with a 50 fold excess of the blocking peptide (D, F, H, J) before proceeding with the immunostaining protocol and compared with immunostaining using antibody alone (C, E, G, I). ALS spinal cord (C, D, G, H) and FTLD frontal cortex (E, F, I, J). Scale bar = 100 um. (PDF) [file pgen.1004803.s007.pdf]

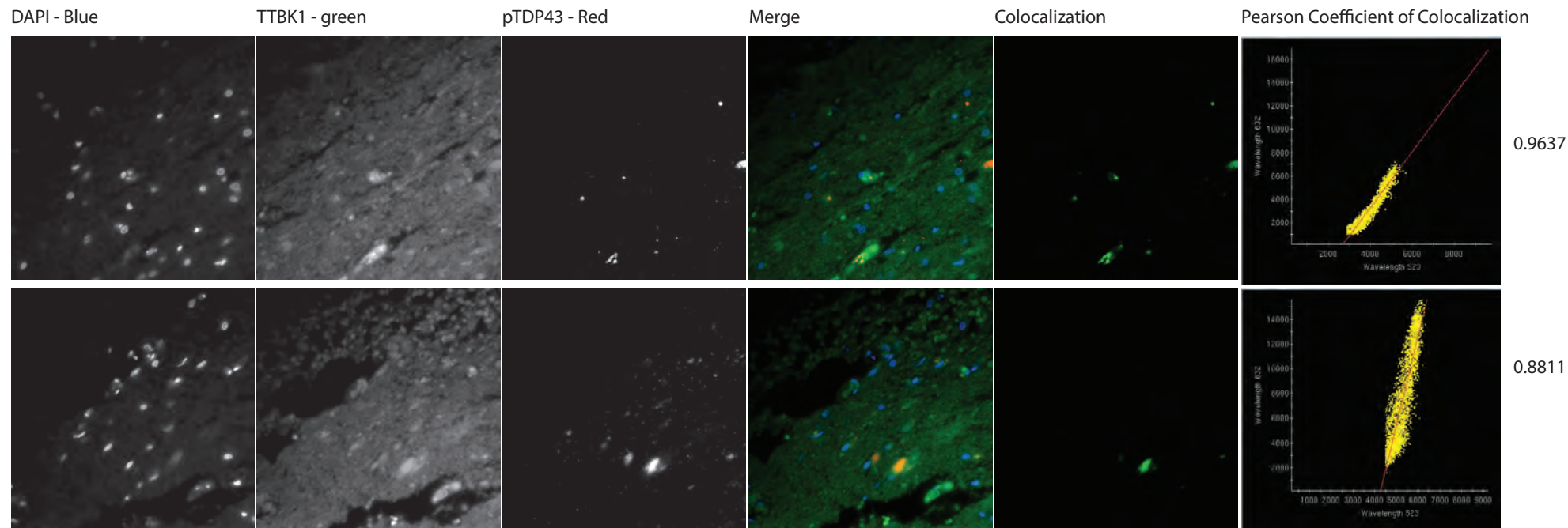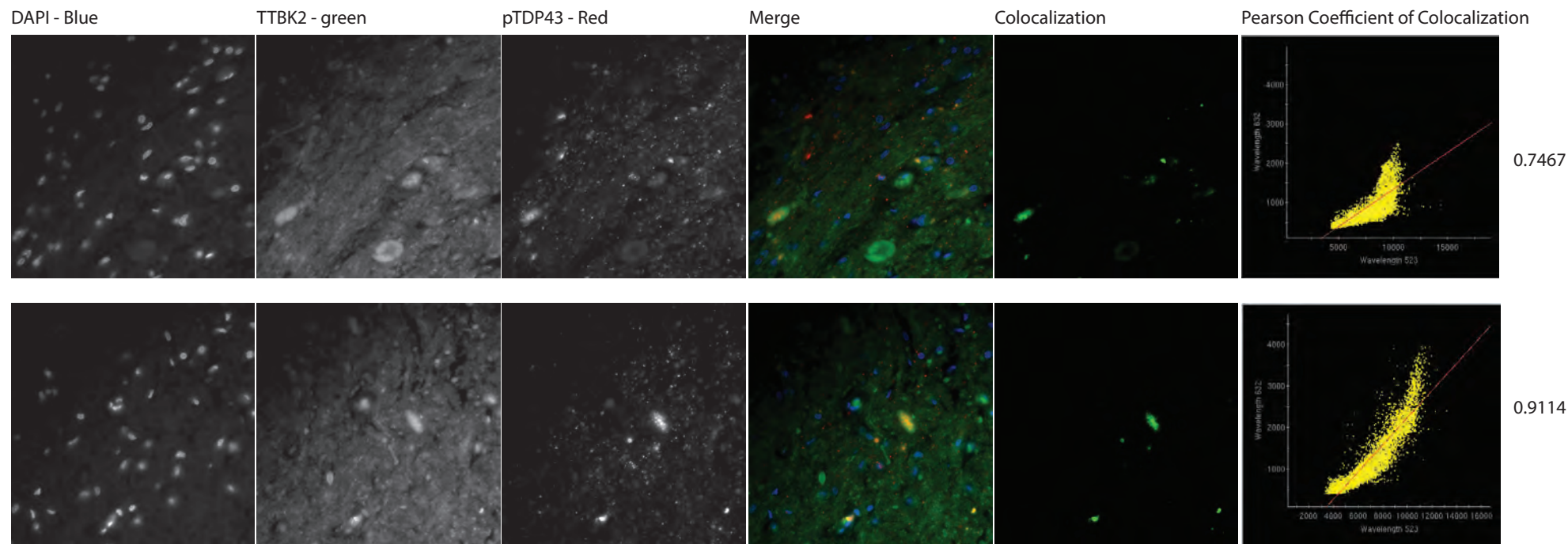

Supplement: S8 Fig — TTBK1/2 co-localize with phosphorylated TDP-43 in aggregates in ALS spinal cord. Double-label immunofluorescence of ALS spinal cord demonstrates additional neurons that significantly co-localize TTBK1 (upper panel) or TTBK2 (lower panel) with phospho-TDP-43 within neuronal cytoplasmic inclusions. Significance was determined using Pearson coefficient of colocalization. (PDF) [file pgen.1004803.s008.pdf]
