# Supplementary material for: The Tau Tubulin Kinases TTBK1/2 Promote Accumulation of Pathological TDP-43
Source: PLoS Genet. 2014 Dec 4;10(12):e1004803. doi: 10.1371/journal.pgen.1004803 (PMC4256087; doi:10.1371/journal.pgen.1004803)
Supplement: S3 Figure — H05L14.1 and dkf-2 mammalian homologs identified by BLAST. (A, B) Cladogram of vertebrate homologs of the C. elegans proteins H05L14.1 and dkf-2. The entire C. elegans amino acid sequences of H05L14.1 or dkf-2 were compared against non-redundant reference sequences from the RefSeq protein database (7-20-2014, NCBI). (A) 2878 active hits were identified by BLAST with similarity to H05L14.1, and subsequently filtered to include only sequences from C. elegans or phylum chordata. The top 50 hits underwent multiple sequence alignment, alignment refinement, phylogenetic reconstruction, and are displayed in a cladogram, with branch support values in red [27]. Related human gene and gene identifier is boxed. Homo sapiens GI# 58761548 is TTBK1. (B) 5000 active hits were identified with similarity to dkf-2, filtered, and graphed as above. Homo sapiens GI# 5031689 is PRKD3. (C) H05L14.1 kinase domain has 40% identity to human TTBK1 and TTBK2. (D) dkf-2 has more than 70% identity to human PRKD2 and PRKD3. Sequence identity was calculated using Clustal W method for multiple sequence alignment. (E) Alignment report for H05L14.1, TTBK1, and TTBK2 kinase domain, including boxes around sequence that matches the consensus. (F) Alignment report for dkf-2, PRKD2, and PRKD3. Reports were generated using Lasergene MegAlign software for protein sequence analysis and alignment. (PDF) [file pgen.1004803.s003.pdf]

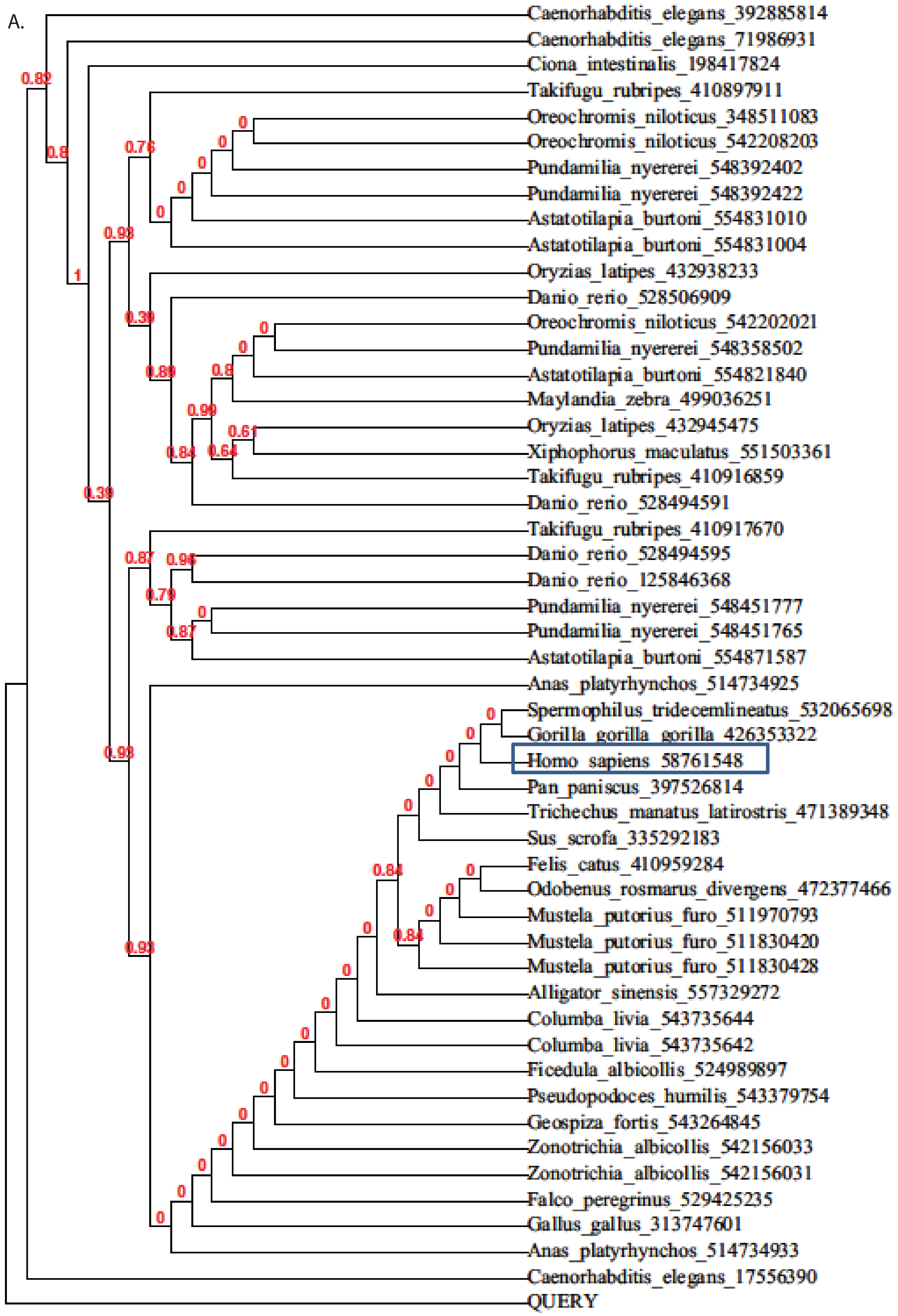
A.
Caenorhabditis_elegans_392885814
Caenorhabditis_elegans_71986931
Ciona_intestinalis_198417824
Takifugu_rubripes_410897911
Oreochromis_niloticus_348511083
Oreochromis_niloticus_542208203
Pundamilia_nyererei_548392402
Pundamilia_nyererei_548392422
Astatotilapia_burtoni_554831010
Astatotilapia_burtoni_554831004
Oryzias_latipes_432938233
Danio_rerio_528506909
Oreochromis_niloticus_542202021
Pundamilia_nyererei_548358502
Astatotilapia_burtoni_554821840
Maylandia_zebra_499036251
Oryzias_latipes_432945475
Xiphophorus_maculatus_551503361
Takifugu_rubripes_410916859
Danio_rerio_528494591
Takifugu_rubripes_410917670
Danio_rerio_528494595
Danio_rerio_125846368
Pundamilia_nyererei_548451777
Pundamilia_nyererei_548451765
Astatotilapia_burtoni_554871587
Anas_platyrhynchos_514734925
Spermophilus_tridecemlineatus_532065698
Gorilla_gorilla_gorilla_426353322
Homo_sapiens_58761548
Pan_paniscus_397526814
Trichechus_manatus_latirostris_471389348
Sus_scrofa_335292183
Felis_catus_410959284
Odobenus_rosmarus_divergens_472377466
Mustela_putorius_furo_511970793
Mustela_putorius_furo_511830420
Mustela_putorius_furo_511830428
Alligator_sinensis_557329272
Columba_livia_543735644
Columba_livia_543735642
Ficedula_albicollis_524989897
Pseudopodoces_humilis_543379754
Geospiza_fortis_543264845
Zonotrichia_albicollis_542156033
Zonotrichia_albicollis_542156031
Falco_peregrinus_529425235
Gallus_gallus_313747601
Anas_platyrhynchos_514734933
Caenorhabditis_elegans_17556390
QUERY

B.

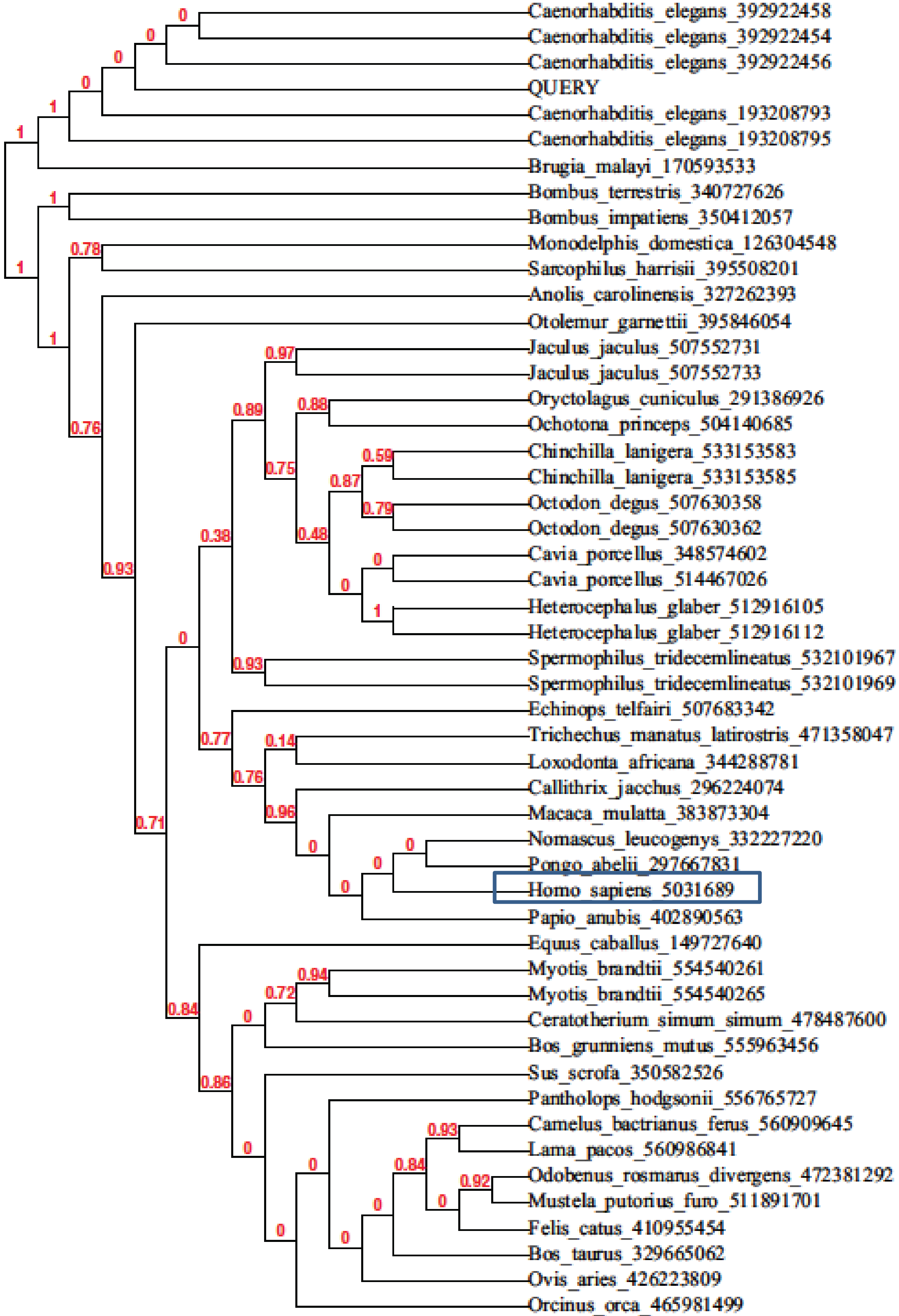

Caenorhabditis_elegans_392922458
Caenorhabditis_elegans_392922454
Caenorhabditis_elegans_392922456
QUERY
Caenorhabditis_elegans_193208793
Caenorhabditis_elegans_193208795
Brugia_malayi_170593533
Bombus_terrestris_340727626
Bombus_impatiens_350412057
Monodelphis_domestica_126304548
Sarcophilus_harrisii_395508201
Anolis_carolinensis_327262393
Otolemur_garnettii_395846054
Jaculus_jaculus_507552731
Jaculus_jaculus_507552733
Oryctolagus_cuniculus_291386926
Ochotona_princeps_504140685
Chinchilla_lanigera_533153583
Chinchilla_lanigera_533153585
Octodon_degus_507630358
Octodon_degus_507630362
Cavia_porcellus_348574602
Cavia_porcellus_514467026
Heterocephalus_glaber_512916105
Heterocephalus_glaber_512916112
Spermophilus_tridecemlineatus_532101967
Spermophilus_tridecemlineatus_532101969
Echinops_telfairi_507683342
Trichechus_manatus_latirostris_471358047
Loxodonta_africana_344288781
Callithrix_jacchus_296224074
Macaca_mulatta_383873304
Nomascus_leucogenys_332227220
Pongo_abelii_297667831
Homo_sapiens_5031689
Papio_anubis_402890563
Equus_caballus_149727640
Myotis_brandtii_554540261
Myotis_brandtii_554540265
Ceratotherium_simum_simum_478487600
Bos_grunniens_mutus_555963456
Sus_scrofa_350582526
Pantholops_hodgsonii_556765727
Camelus_bactrianus_ferus_560909645
Lama_pacos_560986841
Odobenus_rosmarus_divergens_472381292
Mustela_putorius_furo_511891701
Felis_catus_410955454
Bos_taurus_329665062
Ovis_aries_426223809
Orcinus_orca_465981499

C.

Percent Identity

	1	2	3		
1		28.4	30.1	1	**H05L14.1**
2	170.6		90.1	2	**TTBK1**
3	159.6	10.6		3	**TTBK2**
	1	2	3		

D.

Percent Identity

	1	2	3		
1		75.4	71.5	1	**dkf-2**
2	29.9		90.1	2	**PRKD2**
3	35.8	10.6		3	**PRKD3**
	1	2	3		

E.

```
Consensus   WKVLXKIGGGGFGEIYXAXDXLTRENVALKVESAQQPKQVLKMEVAVLKKLQGKDHVCRFIGCGRNXXFNYVVMQLQGRN
                     10        20        30        40        50        60        70        80
H05L14.1    ----------------------------------------------------------LRNIEFCQTQHLNYWISPFSLQ  23
TTBK1       WKVLKKIGGGGFGEIYEAMDLLTRENVALKVESAQQPKQVLKMEVAVLKKLQGKDHVCRFIGCGRNEKFNYVVMQLQGRN  80
TTBK2       WKVLRKIGGGGFGEIYDALDMLTRENVALKVESAQQPKQVLKMEVAVLKKLQGKDHVCRFIGCGRNDRFNYVVMQLQGRN  80

Consensus   LADLRRSQXRGTFTXSTTLRLGXQILESIEXIHSVGFLHRDIKPSNFAMGRLPSTXRKCYMLDFGLARQXTN-STGDVRP
                     90       100       110       120       130       140       150       160
H05L14.1    ILEILR--IVKALTVPCAFNVALQTLDAIQYLHQAGFLNRNIKPASFSVG-LNEEETKIYMTDYRLVRTHIDPSTKKVRP  100
TTBK1       LADLRRSQPRGTFTLSTTLRLGKQILESIEAIHSVGFLHRDIKPSNFAMGRLPSTYRKCYMLDFGLARQYTN-TTGDVRP  159
TTBK2       LADLRRSQSRGTFTISTTLRLGRQILESIESIHSVGFLHRDIKPSNFAMGRFPSTCRKCYMLDFGLARQFTN-SCGDVRP  159

Consensus   PRXVAGFRGTVRYASINAHKNREMGRHDDLWSLFYMLVEFXVG--QLPWRKIKDKEQVGXIKEXYXXXXLXXXXXXXXXX
                    170       180       190       200       210       220       230       240
H05L14.1    ARTGLKYGGTARYASIAGLKKKDQGRKDDVEAWIYMIYDLIDPENGLSWRKSPRCNMMIKEKENFKYHVLPHTYDKVPEE  180
TTBK1       PRNVAGFRGTVRYASVNAHKNREMGRHDDLWSLFYMLVEFAVG--QLPWRKIKDKEQVGMIKEKYEHRMLLKHMPSEFHL  237
TTBK2       PRAVAGFRGTVRYASINAHRNREMGRHDDLWSLFYMLVEFVVG--QLPWRKIKDKEQVGSIKERYD               223

Consensus   FXXXXX----------------
                    250       260
H05L14.1    FKKLVD                                                                            186
TTBK1       FLDHIASLDYFTKPDYQLIMSV                                                            259
TTBK2                                                                                         223
```

F.

```
Consensus   -XXXXXXXXXIQXXXXXSXXYQIFAXEVLGSGQFGXVYGGXHRXXGXXVAXKVIDKLRFPTKQESQLRNEVAILQXLXHP
                     10        20        30        40        50        60        70        80
dkf-2       EGETGHLGAKIQTEHEFSQLYQIFAEEVLGSGQFGTVYGGIHRRNGQHVAVKLIDKLKFPPNKEDLLRAEVQILEKVDHP  80
PRKD2       ---------------------------------------------------KVIDKLRFPTKQESQLRNEVAILQSLRHP  29
PRKD3       -TSISVSNCQIQENVDISTVYQIFADEVLGSGQFGIVYGGKHRKTGRDVAIKVIDKMRFPTKQESQLRNEVAILQNLHHP  79

Consensus   GIVNLECMFETPERVFVVMEKLHGDMLEMILSSEKGRLPERXTKFLVTQILVALRXLHFKNIVHCDLKPENVLLASAXPF
                     90       100       110       120       130       140       150       160
dkf-2       GVVHFMQMLETTDRIFVVMEKLKGDMLEMILSSEKGRLSERTTQFLVAQILEALRYLHHLNIVHCDLKPENILLNSNSDF  160
PRKD2       GIVNLECMFETPEKVFVVMEKLHGDMLEMILSSEKGRLPERLTKFLITQILVALRHLHFKNIVHCDLKPENVLLASADPF  109
PRKD3       GIVNLECMFETPERVFVVMEKLHGDMLEMILSSEKSRLPERITKFMVTQILVALRNLHFKNIVHCDLKPENVLLASAEPF  159

Consensus   PQVKLCDFGFARIIGEKSFRRSVVGTPAYLAPEVLRNKGYNRSLDMWSVGVIXYVSLSGTFPFNEDEDINDQIQNAAFMY
                    170       180       190       200       210       220       230       240
dkf-2       PQVKLCDFGFARIIGEKSFRRSVVGTPAYLAPEVLRNKGFNRSLDMWSVGVIVYVSLSGTFPFNEDEDINDQIQNAEFMY  240
PRKD2       PQVKLCDFGFARIIGEKSFRRSVVGTPAYLAPEVLLNQGYNRSLDMWSVGVIMYVSLSGTFPFNEDEDINDQIQNAAFMY  189
PRKD3       PQVKLCDFGFARIIGEKSFRRSVVGTPAYLAPEVLRSKGYNRSLDMWSVGVIIYVSLSGTFPFNEDEDINDQIQNAAFMY  239

Consensus   PPXPWXEISXXAID--------------------------------------------------
                    250       260       270       280       290       300
dkf-2       PPTPWKEISENAIE                                                    254
PRKD2       PASPWSHISAGAIDLINNLLQVKMRKRYSVDKSLSHPWLQEYQTWLDLRELEGKMGERYITHES  253
PRKD3       PPNPWREISGEAID                                                    253
```
